# The Effect of Tff3 Deficiency on the Liver of Mice Exposed to a High-Fat Diet

**DOI:** 10.3390/biomedicines13051024

**Published:** 2025-04-23

**Authors:** Iva Bazina, Kate Šešelja, Tatjana Pirman, Anita Horvatić, Andreja Erman, Martina Mihalj, Mirela Baus Lončar

**Affiliations:** 1 Faculty of Chemical Engineering and Technology, University of Zagreb, 10000 Zagreb, Croatia; ibazina@fkit.unizg.hr; 2 Division of Molecular Medicine, Ruđer Boškovic Institute, Bjenička 54, 10000 Zagreb, Croatia; kate.seselja@irb.hr; 3 Department of Animal Science, Biotechnical Faculty, University of Ljubljana, Groblje 3, 1230 Domzale, Slovenia; tatjana.pirman@bf.uni-lj.si; 4 Faculty of Food Technology and Biotechnology, University of Zagreb, Pierottijeva 6, 10000 Zagreb, Croatia; anita.horvatic@pbf.unizg.hr; 5 Institute of Cell Biology, Faculty of Medicine, University of Ljubljana, 1000 Ljubljana, Slovenia; andreja.erman@mf.uni-lj.si; 6 Department of Dermatology and Venereology, University Hospital Osijek, 31000 Osijek, Croatia; martina.mihalj@mefos.hr; 7 Department of Physiology and Immunology, Faculty of Medicine, University of Osijek, 31000 Osijek, Croatia

**Keywords:** trefoil factor 3, high-fat-diet treatment, sex difference

## Abstract

**Background/Objectives:** Trefoil factor protein 3 (Tff3) is a small peptide known as an epithelial tissue-protective protein, and it is also identified as a novel participant in complex metabolic processes. In numerous mouse models of obesity, Tff3 has been found to be downregulated in the liver and its overexpression is associated with an improvement in metabolic parameters. These mouse models with metabolic phenotypes have a multigenic background, with numerous genes contributing to their phenotype. To elucidate the role of Tff3 protein in metabolic events, we developed a mouse model with Tff3 deficiency on a C57Bl6N background without other intrinsic mutations affecting metabolism. **Methods**: We investigated the effects of a high-fat diet (9 weeks) on the liver of Tff3 protein-deficient mice of both sexes and the corresponding wild type. We investigated the general metabolic status of the animals and analysed the expression of markers of relevant pathophysiological pathways in the liver. **Results**:Tff3-deficient mice had significantly lower body weight. They also had a comparable total liver fat content but it was distributed in small vesicles, indicating the protective effect of Tff3 deficiency. The results of molecular analysis showed no major gene expression changes in inflammation-, ER- and oxidative stress-, and lipid metabolism-related genes. *Tff3*^−^*^/^*^−^ males had reduced expression of *Il1α* and *Cxcr7* genes in the liver and no global proteome changes; Tff3-deficient females had decreased expression of *Irs2* and *Atf4* genes and total proteome comparison showed decreased levels of proteins related to ribosome biosynthesis and the inhibition of acetylation. **Conclusions**: Our results demonstrate that Tff3 deficiency reduces lipid accumulation in the liver and we set the direction for further studies aimed at uncovering the exact molecular mechanisms in other organs. Furthermore, it emphasises the need to include both sexes in future research, as the observed phenotype differs significantly depending on sex.

## 1. Introduction

A sedentary lifestyle combined with a diet high in fat and carbohydrates but low in fibre contributes to obesity and metabolic syndrome, which are closely linked to conditions previously described as non-alcoholic fatty liver disease (NAFLD) [1]. NAFLD is characterised by the excessive accumulation of lipids, especially triglycerides, which cause the formation of lipid droplets in hepatocytes. Recently, a group of experts agreed to change the nomenclature to MAFLD (metabolic dysfunction-associated fatty liver disease), as such a term better reflects the fact that it is not just a liver disease, but a systemic metabolic disorder that is closely associated with conditions such as insulin resistance, obesity, type 2 diabetes, and cardiovascular disease [2]. Various intrinsic and extrinsic factors are involved in the complex process of maintaining metabolic homeostasis. Recent studies indicate trefoil factor 3 protein (Tff3), as a novel participant in these complex metabolic processes, represents a potential new target for metabolic conditions such as MAFLD and type II diabetes [3,4,5,6,7,8]. Tff3 is a small secretory protein (59 amino acids, 7kDA) and a member of the trefoil factor family of proteins that is studied primarily for its role in the epithelial protection of the gastrointestinal tract and in cancer research [6].

The first link between Tff3 and metabolism emerged from a study using a polygenic mouse model of diabetes and obesity (TallyHo) [3]. Tff3 was found to be transcriptionally active in the liver tissue of healthy C57BL/6 control mice, but was almost absent in the TallyHo diabetes/obesity model. The downregulation of Tff3 expression has also been found in other genetic mouse models of obesity and diabetes (ob/ob, db/db) [4,5] as well as in models of hepatic steatosis [7] and mouse models of diet-induced obesity [4,5]. In addition to the downregulation of Tff3 expression, Tff3 was shown to have a protective effect on metabolic conditions. More precisely, the adenovirus-mediated overexpression of Tff3 in mouse models resulted in improved glucose and insulin tolerance in metabolic tests and the reduced expression of genes involved in gluconeogenesis [4]. Moreover, mice treated with adenovirus (ob/ob, db/db, C57BL/6-DIO) and mice of the B62DF1-DIO strain also showed an improvement in glucose tolerance and a reduction in gene markers for gluconeogenesis in the liver when administered recombinant TFF3 intraperitoneally [8]. Furthermore, Tff3 was shown to bind to peroxisome proliferator-activated receptor alpha (Pparα), thereby increasing its expression [5]. Pparα is an important transcription factor involved in the β-oxidation process of fatty acids in the liver [9]. Treatment with Tff3 resulted in an increase in markers of fatty acid β-oxidation in primary hepatocytes isolated from these mouse models, which was also observed in vivo. When Tff3 expression was restored in the liver using adenoviruses, histological analyses of the liver showed less cell degeneration and fewer lipid droplets compared to control mice. In addition, Tff3 treatment led to an improvement in fatty liver phenotype, accompanied by reduced serum triglycerides, increased ketone content, and increased expression of fatty acid β-oxidation markers [5].

The above-mentioned studies demonstrate the downregulation of Tff3 in liver tissue of mouse models of diabetes and obesity, and the restoration of Tff3 expression has a protective effect on certain metabolic parameters and the pathology of MAFLD. However, *Tff3^−/−^* (C57BL/6J/Sv129) mice fed a standard diet exhibit improved glucose and insulin tolerance compared to their wild-type controls [10]. They also had increased small lipid vesicles in the liver and showed a significant difference in fatty acid composition compared to the wild-type group. In addition to the studies on mouse models, a lower serum TFF3 level was found in patients with type 1 diabetes compared to healthy individuals, which increased significantly after insulin treatment [11]. In contrast, patients with type 2 diabetes and chronic kidney complications had significantly increased TFF3 concentrations in both serum [12] and urine [13].

It is therefore clear that Tff3 plays a role in the relevant metabolic events that affect the liver, but the exact mechanisms still need to be clarified. Previously described mouse models for metabolic diseases (obesity/diabetes) exhibit various polygenic mutations that contribute to the phenotypes of the mouse strains. To understand the effects of a specific protein deficiency, a well-defined genetic composition without additional mutations affecting the phenotype of the mice is required. To further elucidate the role of the Tff3 protein in obesity-related diseases, we have developed a new congenic Tff3-deficient mouse strain on the C57Bl&N (Tff3^−/−^/C57Bl6N) genetic background that has no additional mutations that could affect metabolic pathways [14]. Recently, our research group investigated the effects of a prolonged high-fat diet (8 months) on a new congenic *Tff3^−/−^* (C57BL/6N) mouse model compared to wild-type controls of both sexes [15]. High-fat-diet treatment is standardly used to induce symptoms of metabolic syndrome in animal models, including obesity, a fatty liver phenotype, and impaired glucose homeostasis, insulin, and lipid metabolism [16]. The results showed that a new congenic *Tff3^−/−^* (C57BL/6N) mouse model exhibited a reduced fatty liver phenotype after long-term exposure to a high-fat diet (8 months) [15]. This observation was confirmed by histological, ultrastructural, and high-performance liquid chromatography (HPLC) analyses. In particular, reduced fat accumulation was observed in *Tff3^−/−^* animals of both sexes, although the effect was more pronounced in males. In addition, both male and female *Tff3^−/−^* mice showed reduced expression of the peroxisome proliferator-activated receptor gamma *Pparγ* in the liver.

In this study, we wanted to address the effects of Tff3 deficiency in the early stages of metabolic provocation with a high-fat diet. Novel *Tff3^−/−^/C57Bl6N* and WT (C57Bl6N) mice of both sexes were exposed to high-fat-diet treatment for 9 weeks to investigate how Tff3 deficiency affects the metabolic status of the animals and relevant pathophysiological signalling pathways in the liver. We monitored body weight, glucose and insulin tolerance and analysed the blood serum. In the liver tissue, we determined the fatty acid content, the expression of various genes involved in disease-related signalling pathways, and the total proteome difference. Due to the known importance of sex variables in metabolic regulation and the growing awareness of the need to include both sexes in biomedical research on various human diseases [17], we included both males and females in this study.

## 2. Materials and Methods

### 2.1. Animals and Diet Treatment

A new congenic trefoil factor family 3-deficient (*Tff3^−/−^*) mouse strain on a C57BL/6N (Charles River, Vilmington, MA, USA) genetic background was developed from a mixed-background strain (C57BL/6J/SV129), as previously described [14]. The new congenic *Tff3^−/−^*C57BL/6N mice and the corresponding wild-type C57BL/6N control groups of both sexes (at the age of 11 weeks) were subjected to a high-fat diet (58% fat source, lard, hydrogenated palm and soybean oil; 24% carbohydrate; 18% protein; Mucedola, Milano, Italy) for a period of 9 weeks. The control groups were fed a standard diet (4RF21, Mucedola) for a period of 20 weeks. Each group consisted of 10 animals. The mice were raised at the Facility for Laboratory Animals, Ruđer Bošković Institute, as part of the project “Tff3 protein at intersection of metabolism and neurodegeneration” (HRZZ-IP-2016-06-2717), funded by the Croatian Science Foundation. Animal breeding and all experimental procedures were carried out in accordance with the Animal Welfare Law (NN 102/17, 32/19) and the Ordinance on the Protection of Animals Used for Scientific Purposes (NN 55/13, 39/17, 116/19). The research was approved by the Bioethics Committee of the Ruđer Bošković Institute and by the National Council of the Ministry of Agriculture of the Republic of Croatia (CLASS: UP/I-322-01 /19-01 /14 RN: 525-10/025519-3 Zagreb, 5 July 2019, and CLASS: UP/I-322-01/16-01/81, RN: 525-10/0543-21-6 Zagreb, 18 February 2021). The animals were kept under standard conditions, i.e., a 12 h light–dark cycle, a temperature of 22 °C, and a humidity of 60%.

### 2.2. Metabolic Tests

The intraperitoneal glucose tolerance test (IPGTT) was performed according to the protocol of the International Mouse Phenotyping Resource of Standardized Screens [18] on animals fed a standard diet at 9 weeks of age before HFD treatment and at 17 weeks of age after 6 weeks of HFD treatment. The mice were fasted for 16 h, with water available ad libitum; they were then administered 2 g/kg glucose in sterile 1× PBS intraperitoneally. Blood glucose levels from the tail vein blood were measured before glucose administration and 15, 30, 60 and 120 min after glucose administration.

The intraperitoneal insulin tolerance test (IPITT) was performed, following the guidelines of the National Mouse Metabolic Phenotyping Center [19], on animals fed a standard diet at 9 weeks of age before HFD treatment and at 18 weeks of age after 7 weeks of HFD. The test was carried out after a 4 h fasting period with water available ad libitum. Then, 0.75 IU/kg insulin in sterile 1× PBS was injected intraperitoneal. Blood glucose levels from the tail vein blood were measured immediately before insulin administration and 15, 30, 60, 45 and 120 min after administration.

### 2.3. Blood Biochemistry

Blood samples (n = 5) from the jugular vein were collected at the time of sacrifice and left at room temperature to allow the blood to clot. Subsequently, they were centrifuged at 4 °C and 1000× *g* for 10 min and the supernatant serum was collected into clean tubes. Serum levels of low-density lipoprotein (LDL), high-density lipoprotein (HDL), total cholesterol, triglycerides (TGs), urates, urea, total proteins (TPs), aspartate transaminase (AST), alanine transaminase (ALT), uric acid, and alkaline phosphatase (ALP) were determined using an Abott Architect c8000 clinical chemistry analyser and appropriate IVD clinical chemistry kits (Abbot, Chicago, IL, USA).

### 2.4. Oil Red O Staining of Lipids in Liver Tissue

The mice were sacrificed at 20 weeks of age after receiving HFD for 9 weeks. Half of them (n = 5) were fixed by transcardial whole-body perfusion with 4% paraformaldehyde (PFA). Liver tissue was harvested and stored in fresh 4% PFA. It was then incubated overnight in a 15% sucrose solution (Carl Roth, GmbH, Karlsruhe, Germany) in 1× PBS and then in 30% sucrose. To ensure gradual freezing, the tissue was frozen in moulds filled with embedding matrix (OCT Embedding Matrix, Carl Roth, GmbH). These were placed in liquid 2-methylbutane (VWR, West Chester, PA, USA) and precooled to −80 °C. The embedded tissues were stored at −80 °C until further processing. The samples were sectioned to a thickness of 8 µm using a cryotome (Leica CM 3050S cryotome, Wetzlar, Germany). The cryosections were mounted on glass slides (SuperFrost^®^, VWR). A 0.5% solution of Oil Red O dye (Sigma Aldrich, St. Louis, MO, USA) in 2-propanol was diluted with deH_2_O at a ratio of 3:2, followed by filtration. Filter paper soaked in 10% formalin was placed in a glass slide container and the slides containing the liver cryosections were placed in the container at 4 °C for 5 min. They were then briefly immersed in 60% 2-propanol and incubated in Oil Red O working solution for 15 min at room temperature in the dark. The slides were washed twice briefly in 60% 2-propanol and then rinsed in deH_2_O. The nuclei were stained with haematoxylin (Hematoxylin, Mayer’s, DakoCytomation, Glostrup, Denmark) for 1 min, after which the slides were rinsed again in deH_2_O. Cryosections were mounted with mounting medium (ROTI^®^Mount Aqua, Carl Roth) and analysed using an Olympus BX51TF microscope and Olympus Stream Essentials software v2.4 (Olympus, Tokyo, Japan).

### 2.5. Analysis of the Fatty Acid Composition

Liver tissue from the other half of the mice (n = 5), sacrificed at 20 weeks of age, was harvested, snap-frozen in liquid nitrogen, and stored at −80 °C for future use. The fatty acid composition of the liver was analysed by gas chromatography. Briefly, 0.25 g of the homogenised sample was transmethylated in situ using 0.5 M NaOH in methanol. This was followed by the placement of 14% BF3 (boron trifluoride) in methanol according to the Park and Goins method [20]. Fatty acid methyl esters (FAMEs) were extracted with hexane. We used an Agilent 6890 GC equipped with a DB-Fatwax UI chromatography column (30 m length; 0.25 mm i.d., 0.25 m film thickness; Agilent Technologies, Santa Clara, CA, USA) and an FID detector for FAME separation.

### 2.6. qPCR Analysis

Total RNA was isolated from the frozen liver tissue of male and female Tff3^−/−^ and WT mice (n = 5) using an NucleoSpin RNA (MACHEREY-NAGEL, Düren, Germany) kit following the manufacturer’s instructions. The transcription of the RNA into cDNA was performed using the high-capacity cDNA reverse transcription kit (Applied Biosystems, Dreieich, Germany). A quantitative polymerase chain reaction (qPCR) was carried out on the StepOne™ Real-Time PCR System (Applied Biosystems) utilising SYBR Green (Thermo Fisher Scientific, Waltham, MA, USA) detection chemistry. PCR cycling conditions included three-minute polymerase activation at 95 °C, followed by 40 amplification cycles, each of which included 1 min of denaturation at 95 °C, 30 s of annealing at a specific temperature for each primer pair (Table S1), and 30 s of DNA elongation at 72 °C. The amplification of a single product was confirmed by melting curve analysis and polyacrylamide gel electrophoresis. Gene expression was normalised to β-actin (Actβ) and β2-microglobulin (β2m). The relative expression of genes is presented as fold change.

### 2.7. LC-MS/MS Analysis

Total protein was isolated from the liver tissue of WT and Tff3^−/−^ mice (n = 5), fed a high-fat diet, using the Minute TM Total Protein Extraction Kit for Animal Cultured Cells/Tissues (Invent Biotechnologies, Plymouth, MN, USA), according to the manufacturer’s instructions, and protein concentration was determined using the Pierce BCA Protein Assay Kit (Thermo Fisher Scientific). High-resolution liquid chromatograph tandem mass spectrometry (LC-MS/MS) analysis was performed for protein identification and relative quantification. Samples were labelled with tandem mass tags (TMT tenplex reagents; Thermo Scientific), and an internal standard was prepared. Dried TMT-labelled peptides were dissolved in a loading buffer (2% ACN in 0.1% FA) and 1 μg was desalted on the trapping column using the Ultimate 3000 RSLCnano system (Dionex, Germering, Germany). Peptides were then separated using the PepMap™ RSLC C18 column (50 cm × 75 μm ID) during a 2 h linear gradient of 5–35% buffer B (0.1% FA in 80% ACN) at a flow rate of 300 nL/min. A Nanospray Flex ion source and a stainless-steel emitter (New Objective, Woburn, MA, USA) were used. The ionisation voltage was set to 2.1 kV and the temperature of the ion transfer tube was 250 °C. DDA was performed in positive ion mode according to the Top 8 method using Q Exactive Plus (Thermo Scientific, Bremen, Germany). Full-scan FTMS spectra were acquired in a mass range from *m*/*z* 350.0 to *m*/*z* 1900.0 with the following conditions: a resolution of 70,000, an AGC target of 1 × 10^6^, and a maximum injection time of 110 ms. For MS/MS scan, step collision energy was set to 25, 35, and 40% NCE with a resolution of 17,500 and an AGC target of 2 × 10^5^. An isolation window of ±2.0 Da with a dynamic exclusion of 30 s was applied to isolate precursor ions. 

### 2.8. Western Blot

The total protein from liver tissue (n = 5) was isolated with a RIPA buffer (50 mM TRIS HCL, pH8, 150 mM NaCl, 1 mM EDTA, 1% NP40, 1% sodium deoxycholate, 0.1% SDS), supplemented with phosphatase and protease inhibitors. The protein concentration was determined using the BCA protein assay kit (Pierce, Thermo Fisher, Waltham, MA, USA), and we separated 10 µg of proteins per lane by sodium dodecyl sulphate–polyacrylamide gel electrophoresis (SDS-PAGE). Proteins were transferred to a PVDF membrane for 16 h at 4 °C using a constant current of 100 mA. Then, a 5% I-Block™ protein-based blocking reagent (T2015) in 1× PBS was used to block non-specific binding for 1 h. Membranes were incubated overnight at 4 °C with anti-Ybx1 (ab76149, Abcam, Cambridge, UK) and anti-Anp32a primary antibodies (15810-1-AP, Proteintech, Manchester, UK), diluted 1:1000. The membrane was then washed 3 × 10 min with a 1× TBS-T buffer and incubated with the secondary antibody goat anti-rabbit IgG-HRP (#170-6515, Bio-Rad Laboratories, Hercules, CA, USA). The same washing steps were repeated before the specifically labelled proteins were visualised using Pierce™ ECL Western Blotting Substrate (Thermo Fisher Scientific) according to the manufacturer’s instructions and imaged using the Uvitec Alliance Q9 Mini Chemiluminescence Imaging System. The normalisation of the bands was performed by Amido Black staining. The quantification of proteins was performed using ImageJ software (https://imagej.net, accessed on 6 April 2025).

### 2.9. Statistical Analyses

Body weight measurements and blood glucose values from IPGTT and IPITT were analysed by two-way ANOVA, followed by a Tukey post hoc analysis with multiple comparisons. The results of the biochemical analysis of blood sera were also analysed by two-way ANOVA, followed by the Bonferoni post hoc test. Liver fat and fatty acid content were analysed using general linear models (GLMs) of the SAS/STAT module (SAS Institute Inc., Cary, NC, USA), with the differences determined by the Tukey–Kramer post hoc test, with genotype as the main effect. This was performed separately for male and female mice. Relative gene expression was determined using REST MCS © software v2 (ΔΔCt method) and normalised to stable housekeeping genes β-actin (*Actβ*) and β2-microglobulin (*β2m*). The SEQUEST algorithm implemented in Proteome Discoverer v 2.3 software (Thermo Fisher Scientific) and the Mus musculus FASTA file database search (NCBI database) were used for proteomic data analysis and protein identification. To obtain reliable protein identification, at least two unique peptides and a 5% false discovery rate (FDR) threshold were set. The FDR for the entire dataset was calculated based on the results of the database search using the Percolator algorithm in the Proteome Discoverer programme. The internal standard was used as a reference sample. Normalisation of occurrence was based on the total protein amount and the protein ratio calculation. A study with biological replicates was performed using the so-called non-nested design without entering missing values. The unpaired t-test was used to calculate the *p*-value and then the Benjamini–Hochberg correction with a *p*-value of 0.05 was applied. The results of protein detection by Western blot were quantified using ImageJ software and the *t*-test was used to determine the differences between the obtained mean values of protein levels of two independent groups. All graphical representations were created using GraphPad Prism version 8.0.0 (GraphPad Software, La Jolla, CA, USA).

## 3. Results

### 3.1. Body Weight and Metabolic Parameters

**Figure 1:**
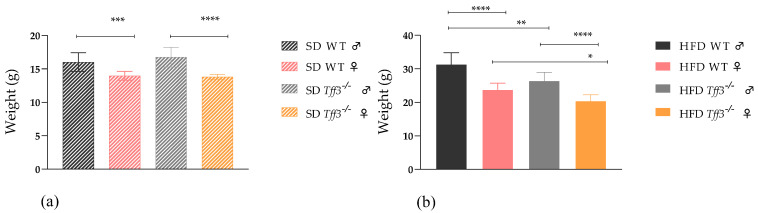
The effect of Tff3 deficiency on the body weight of mice of both sexes fed with a SD and an HFD: (**a**) weight of 9-week-old mice fed with an SD; (**b**) weight of 20-week-old mice after 9 weeks of HFD treatment; n ~ 10 * *p* ≤ 0.05, ** *p* ≤ 0.01, *** *p* ≤ 0.001,**** *p* ≤ 0.0001. Symbols: ♂—male; ♀—female.

Body weight was measured in WT and *Tff3^−/−^* of both sexes, at age of 9 weeks, before the high-fat-diet treatment (HFD). In other words, the weight of 9-week-old animals fed a standard diet (SD) was measured (Figure 1a). There were no significant differences in body weight between WT and *Tff3^−/−^* animals when fed SD, but after 9 weeks of HFD, both *Tff3^−/−^* males and females weighed less than WT controls (Figure 1b). As expected, female mice weighed less than male mice, and this was measured in both SD- and HFD-fed animals (Figure 1a,b).

In addition to weight measurements, various biochemical parameters were analysed in the serum of mice (total cholesterol, HDL, LDL, triglycerides, liver enzymes ASP, ALT and AST, total proteins, urea, and urates). These parameters can point to deregulations of lipid metabolism and indicate liver and kidney dysfunctions. The only statistically significant difference related to the genotype was the reduced level of triglycerides in the serum of *Tff3^−/−^* males compared to WT males (Figure S1).

Intraperitoneal glucose and insulin tolerance testing (IPGTT, IPITT) were performed to examine metabolic health of animals. IPGTT was performed on 9-week-old animals before exposure to an HFD (Figure S2). It was shown that *Tff3^−/−^* males have a higher concentration of glucose in the blood, but only 30 min after the injection of glucose compared to WT males (Figure S2a). *Tff3^−/−^* females had a lower blood glucose concentration 15 and 30 min after the initial injection compared to WT females (Figure S2b). IPGTT was again performed after 6 weeks of HFD treatment (Figure S3). The differences observed in animals on SD were not detected after 6 weeks of exposure to an HFD (Figure S3a,b). IPITT was performed on 9-week-old animals before exposure to an HFD (Figure S4) and after 7 weeks of an HFD (Figure S5). Animals on a SD do not show statistically significant differences in blood glucose concentration after insulin injection (Figure S4). However, after 7 weeks of HFD, *Tff3^−/−^* males have significantly lower glucose levels compared to WT males at almost all time points (Figure S5a), while there are no detectable statistically significant differences in *Tff3^−/−^* females compared to WT females (Figure S5b).

### 3.2. Liver Lipid Analysis

**Figure 2:**
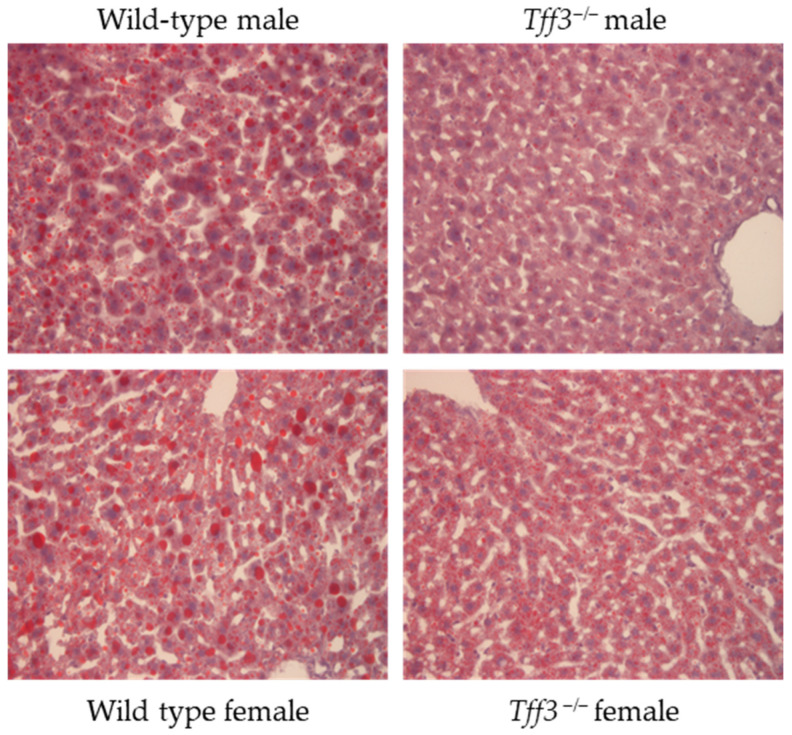
Lipid visualisation by Oil Red O staining in liver cryosections of both male and female WT and *Tff3^−/−^* after 9 weeks. Oil Red O is used for staining lipids (red) and nuclei are stained with haemoxylin (purple): Scale: 200 µm.

The accumulation of neutral lipids in the liver was analysed by Oil Red O staining of cryosections from both WT and Tff3^−/−^ animals after short-term HFD exposure (Figure 2). Both male and female Tff3^−/−^ mice showed fewer lipid vesicles stained with Oil Red O compared to WT mice.

To further investigate the observed effects, we analysed the specific content of the major fatty acids in the liver (Table 1) of HFD-fed Tff3^−/−^ and WT mice. Total fat content was not statistical changed in Tff3-deficient mice of both sexes. Tff3^−/−^ males had a significantly lower content of arachidic acid (C20:0) compared to WT males. The content of various fatty acids was altered in the liver of Tff3^−/−^ females compared to WT females, so that they had a higher content of linoleic acid (C18:2, n-6), linolenic acid (C18:3, n-6), eicosapentaenoic acid (C20:5, n-3), and docosapentaenoic acid (C22:5, n-3), while the content of gadoleic acid (C20:1, n-9) was lower.

In addition, compared to WT females, *Tff3^−/−^* females had significantly higher total levels of omega-3 polyunsaturated fatty acid content, and the ratio of omega-6 to omega-3 polyunsaturated fatty acids was significantly lower in the livers of *Tff3^−/−^* females.

As expected, there were statistically significant differences in fatty acid levels between the sexes. WT females have lower levels of arachidic acid (C20:0), dihomo-γ-linolenic acid (C20:3, n-6), eicosapentaenoic acid (C20:5, n-3), and docosapentaenoic acid (C22:5, n-3) in the liver compared to WT males. Differences were also observed in Tff3^−/−^ females compared to Tff3^−/−^ males. Thus, the content of arachidonic acid (C20:0), gadoleic acid (C20:1, n-9) and dihomo-γ-linoleic acid (C20:3, n-6) was lower, while the content of docosapentaenoic acid (C22:5, n-3) was higher. Tff3^−/−^ female mice had an increased ratio of omega-6 to omega-3 polyunsaturated fatty acids compared to Tff3^−/−^ male mice.

### 3.3. Expression of Genes Related to Relevant Pathophysiological Pathways

First, the gene expression of Tff3 was analysed in the liver of 20-week-old C57BL6/N wild-type mice (Figure S6). The aim was to identify possible differences in the expression of Tff3 in the liver in relation to the sex of the animals and possible changes due to treatment with a high-fat diet. The results show that the expression of the Tff3 gene in the liver of females is significantly lower than that of males (Figure S6a,b) and that Tff3 gene expression in the liver is not significantly altered by the treatment (Figure S6c,d).

**Figure 3:**
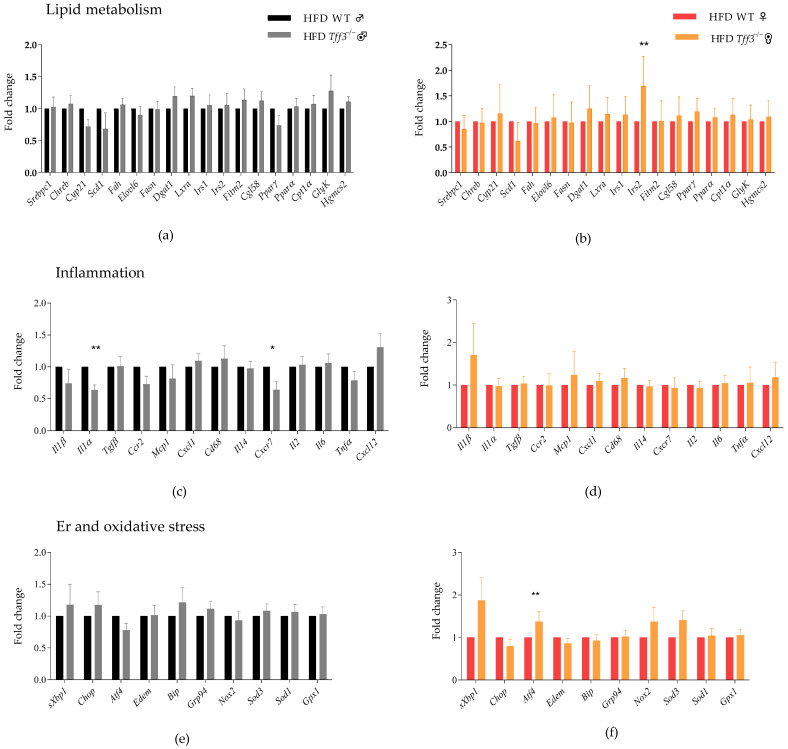
Gene expression of markers involved in lipid metabolism, inflammation, and endoplasmatic reticulum (ER) and oxidative stress in liver of WT and Tff3^−/−^ mice exposed to high-fat diet (HFD) for 9 weeks. Mice were sacrificed, liver tissue was collected, RNA was isolated, and qPCR method based on Sybr green detection was used for gene expression (n = 5 mice per group). Ct values obtained were analysed using software REST©, and results are presented in form of fold change mean and standard error of mean (SEM) with appropriate group expression set to 1: (**a**) lipid metabolism—Tff3^−/−^ males compared to WT males (WT male = 1); (**b**) lipid metabolism—Tff3^−/−^ females compared WT females (WT female = 1); (**c**) inflammation—Tff3^−/−^ males compared to WT males (WT male = 1); (**d**) inflammation—Tff3^−/−^ females compared WT females (WT female = 1); (**e**) ER and oxidative stress—Tff3^−/−^ males compared to WT males (WT male = 1); (**f**) ER and oxidative stress—Tff3^−/−^ females compared WT females (WT female = 1). Statistical significance is shown by * *p* ≤ 0.05, ** *p* ≤ 0.01. Symbols: **♂**—male; **♀**—female.

In addition, the comprehensive analysis of genes involved in relevant pathophysiological processes of metabolic disorders, which are also associated with the effect of the Tff3 peptide, was carried out using the qPCR method. Markers of lipid metabolism, inflammation, endoplasmic reticulum stress, and oxidative stress were analysed (Figure 3). Gene expressions were compared between WT and Tff3^−/−^ of both sexes in liver tissue from animals exposed to short-term treatment with an HFD. For genes involved in lipid metabolism (e.g., de novo lipogenesis, β-oxidation of fatty acids, insulin signalling, gluconeogenesis), there were no detectable statistically relevant differences in the liver of Tff3^−/−^ males compared to WT males, while the insulin receptor substrate 2 (Irs2) gene was upregulated in the liver of Tff3^−/−^ females compared to WT females (Figure 3b). The analysis of cytokines and chemokines involved in the development of MAFLD revealed that interleukin Il1α (Il1α) and atypical chemokine receptor 3 (Cxcr7) were downregulated in the liver of Tff3^−/−^ males compared to WT males, whereas no statistically significant differences were detected in Tff3^−/−^ females (Figure 3c,d).

There were no significant differences in the gene expression of endoplasmic reticulum stress and oxidative stress markers in the liver of Tff3^−/−^ males compared to WT males (Figure 3e), but the level of activating transcription factor 4 (Atf4) gene was upregulated in the liver of Tff3^−/−^ females compared to WT females (Figure 3f).

To investigate whether Il1α, Cxcr, Atf4, and Irs2 expression were already altered under standard conditions due to genotypic differences or whether the changes occurred with exposure to HFD, -qPCR analysis of livers from animals fed an SD was performed. In liver tissue from control animals exposed to a standard diet, there were no statistically significant changes in the expression of the markers analysed, suggesting that the change in expression occurs as a consequence of treatment with a high-fat diet (Figure S7).

### 3.4. LC MS/MS Analysis of Liver in WT and Tff3^−/−^ Mice Exposed to Short-Term HFD Treatment

**Figure 4:**
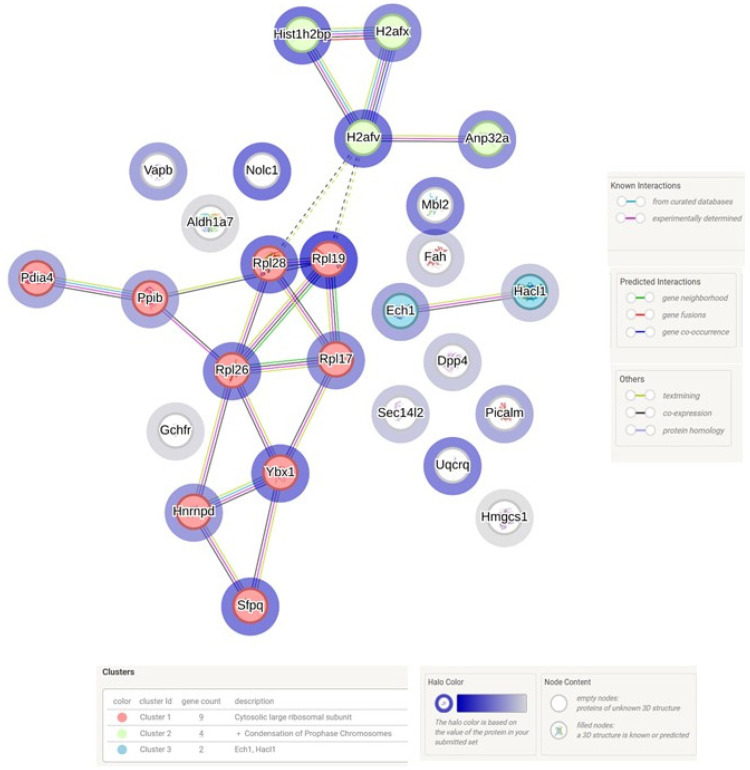
Visualization of protein interactions that were statistically significantly altered in livers of Tff3^−/−^ females compared to WT females fed on high-fat diet. Interactions were visualised using programme tool STRING [21]. Relative expression changes are represented by blue circle around protein (gradation from upregulated (dark blue) to downregulated level (pale blue circle). Abbreviation: Aldh1a7—aldehyde dehydrogenase, cytosolic 1; Anp32a—acidic nuclear phosphoprotein pp32; Dpp4—dipeptidyl peptidase 4 isoform 1; Ech1—enoyl coenzyme A hydratase 1, peroxisomal; Fah—fumarylacetoacetase hydrolase; Gchfr—GTP cyclohydrolase I feedback regulator; H2afv—histone H2afv; H2afx—histone H2A.X; Hacl1—2-hydroxyacyl-CoA lyase 1; Hist1h2bp—histone H2B type 1-P isoform 1; Hmgcs1—3-hydroxy-3-methylglutaryl-coenzyme A synthase 1; Hnrnpd—heterogeneous nuclear ribonucleoprotein AU-rich element RNA-binding protein 1; Mbl2—mannose-binding protein C precursor; Nolc1—nucleolar and coiled-body phosphoprotein 1; Pdia4—protein disulfide-isomerase A4 isoform 1 precursor; Picalm—phosphatidylinositol binding clathrin assembly protein; Ppib—peptidylprolyl isomerase B; Rpl17—ribosomal protein; Rpl19—ribosomal protein L19; Rpl26—60S ribosomal protein L26; Rpl28—60S ribosomal protein L28 isoform X1; Sec14l2—SEC14-like protein 2; Sfpq—splicing factor proline/glutamine-rich polypyrimidine tract binding-associated protein; Uqcrq—ubiquinol-cytochrome c reductase, complex III subunit VII; Vapb—vesicle-associated membrane protein, associated protein B and C; Ybx1—Y-box binding protein.

Proteomic analysis was used to compare the difference in the livers of WT and Tff3^−/−^ mice exposed to a high-fat diet. The application of the false discovery rate (FDR) tool, which compensates for large datasets, revealed no relevant statistical changes in the expression of liver proteins in male Tff3^−/−^ mice compared to male WT mice. Interestingly, female Tff3-deficient mice had 28 proteins with significantly altered expression in the liver compared to WT mice (Table 2). The STRING programme was used to visualise possible protein interactions, as shown in Figure 4. STRING analysis identified four protein networks, one of which included the ribosomal proteins (60s ribosomal protein L17 (Rpl17), 60s ribosomal protein L19 (Rpl19), 60s ribosomal protein L26 (Rpl26), 60s ribosomal protein L28 (Rpl28), and small ribosomal subunit protein S13 (Rsp13)) and their interaction with the Y-box binding protein (Ybx1), heterogeneous nuclear ribonucleoprotein D0 (Hnrnpd), splicing factor, and proline- and glutamine-rich protein (Sfpq), which are upregulated in the liver of Tff3^−/−^ females compared to WT females. The second network includes proteins from the histone family (histone h2b type 1-P (Hist1h2bp), histone h2a.v (H2afv), and histone h2ax (H2afx)) and acidic leucine-rich nuclear phosphoprotein 32 family member A protein (Anp32a), which are also upregulated in the liver of Tff3^−/−^ females compared to WT females.

#### Western Blot Analysis of Ybx1 and Anp32a Protein Expression

**Figure 5:**
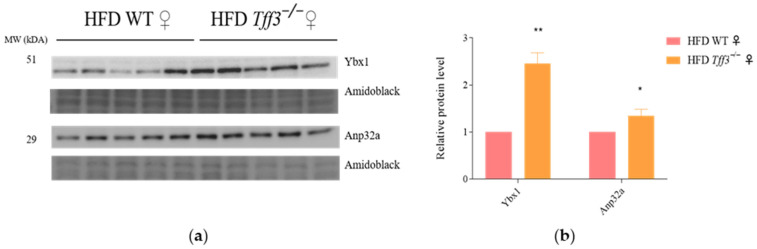
Ybx1 and Anp32a protein levels were significantly upregulated in the liver of Tff3^−/−^ females compared to WT female. Proteins were isolated from the liver tissue of mice exposed to a high-fat diet and analysed by Western blotting. (**a**) The protein level of Ybx1 and Anp32a in the liver of WT and Tff3^−/−^ females exposed to high-fat diet (HFD) analysed by the Western blot method using specific antibodies. (**b**) Results were quantified using the Image J programme, and signals were normalised using amidoblack staining. Student’s *t* test was used for statistical analysis of the obtained values and the results are presented as the mean value and standard deviation. Statistical significance is shown: * *p* ≤ 0.05, ** *p* ≤ 0.01. Symbols: ♀—female.

Proteomic analysis showed the upregulation of Ybx1 and Anp32a proteins in the liver of Tff3^−/−^ females compared to WT females; as such, the Western blot method was used to further validate results. We confirmed the significant upregulation of Ybx1 and Anp32a protein in the liver of Tff3^−/−^ females compared to WT females (Figure 5).

## 4. Discussion

Various studies have shown that Tff3 can play an important role in metabolic processes in the liver [3,4,5,7,8,22]. The fatty liver phenotype in animal models is usually induced by treatment with a high-fat diet. Even short-term exposure to this form of nutritional stress is sufficient to activate the pathological features of metabolic syndrome such as impaired lipid metabolism in the liver and deregulated insulin and glucose metabolism [23]. As it is important to find therapeutic solutions in the early stages of the pathogenesis of these complex diseases before advanced symptoms appear, we exposed WT and Tff3-deficient mice to 9 weeks of HFD treatment and observed the effects on various features of metabolic disorders, including insulin and glucose metabolism, the accumulation of lipids in the liver, and underlying molecular alterations. The aim was to investigate possible differences related to Tff3 deficiency in these processes.

Previous studies found the overexpression of TFF3 in obese mice, while our Tff3-deficient mice fed an HFD also showed a reduction in liver steatosis. The discrepancy in the results may be due to several factors mentioned earlier, such as the genetic background of the strain, the duration and type of treatment, and the age and sex of the animals, which affect metabolic traits and thus emphasise the complexity of the regulation of lipid metabolism. However, the main difference between the studies mentioned above, in which Tff3 was shown to have a positive effect on the metabolic phenotype (in this case it was a reduction in fat accumulation in the liver), and the results of our research group, in which Tff3 deficiency led to a protective effect, was the experimental model. It appears that the targeted regulation of Tff3 expression by adenoviral overexpression (it is hypothesised that this occurs in the liver) has a positive effect on the metabolic phenotype and protects against fat accumulation in the liver caused by a high-fat diet [4,5,8], while the absence of Tff3 in the whole organism (whole-body knock-out model) indicates the same. As expected, this indicates the existence of an interaction between different organs.

It is important to emphasise that we used a newly developed congenic Tff3^−/−^ mouse strain [14] developed on the C57BL/6N background, with no other relevant mutations affecting the metabolic phenotype. Furthermore, as it is known that there are differences in metabolic regulation between males and females that may influence the development of metabolic disorders and cause differential responses to pharmacological interventions [17], animals of both sexes were included in the studies.

### 4.1. Body Weight and Metabolic Parameters

Tff3-deficient mice (male and female) fed on the standard diet (20 weeks old) showed no significant differences in body weight compared to WT animals of the same sex (Figure 1a). However, after a 9-week high-fat diet, both male and female Tff3^−/−^ mice weighed less than WT controls (Figure 1b). Tff3^−/−^ males had a statistically significant lower body weight. Females showed the same trend, but without statistical significance. A similar trend was observed in our previous studies in which the same mouse models were exposed to a long-term HFD [15]. The new congenic Tff3^−/−^ (C57BL/6N) mouse strain was observed from weaning to two years of age under standard dietary conditions, and its body weight was similar to that of the WT (C57BL/6N) strain. In a previous study using a mixed *Tff3^−/−^* (C57BL6/J/129Sv) background strain, no weight differences were found between standard-fed *Tff3^−/−^* mice and WT controls [10], while in another study, a significantly lower weight was found compared to the WT [24]. In the study in which no differences were found, 12-week-old male mice were used, while in the study in which the difference was found, only four 22-week-old animals were used, the sex of which was not specified. Various factors, such as strain, age, sex, and type of feeding, have a significant influence on the weight of the animals, so these complex interactions could explain the discrepancies in the results.

However, we can say that we mainly observed that Tff3 deficiency had no effect on body weight when the animals were fed a standard diet, but when the stress factor caused by a high-fat diet occurred, the animals lacking the Tff3 protein seemed to be partially protected from weight gain.

In addition to weight measurements, biochemical parameters (total cholesterol, HDL, LDL, triglycerides, liver enzymes ALP, ALT and AST, total proteins, urea, and urates) were analysed in the sera of the animals (Figure S1). *Tff3^−/−^* males had downregulated levels of triglyceride compared to WT males, suggesting that Tff3 deficiency could have a protective effect since it is known that elevated level of triglycerides in the serum can indicate the development of metabolic conditions, such as type 2 diabetes. Liver enzymes (ASP, ALT, AST) that indicate liver damage were not significantly changed in the serum of *Tff3^−/−^* mice compared to WT mice after a short-term high-fat-diet treatment (Figure S1). However, they were significantly changed when the same mice models were exposed to a long-term high-fat diet, where Tff3^−/−^ male mice had significantly lower levels of ALP and ALT compared to WT, which suggests the protective effect of Tff3 deficiency against liver damage. The observed reduction in triglycerides suggests the possible protective effect of Tff3 deficiency, but the underlying mechanisms remain speculative.

It is possible that 9 weeks was not long enough to detect the same effect.

Glucose (IPGTTs) and insulin tolerance tests (IPITTs) were performed before the high-fat-diet treatment (Figures S2 and S4) and again after 6 (IPGTT) (Figure S3) and 7 (IPITT) weeks (Figure S5) of treatment. IPGTTs and IPITTs are used to test glucose/insulin tolerance by measuring the blood glucose level after initial glucose/insulin injection. The measurement results provide insight into the metabolic state of the organism and indicate possible irregularities in glucose homeostasis and insulin sensitivity. *Tff3^−/−^* males fed with a standard diet had upregulated glucose levels compared to WT males after 30 min (Figure S2a), while *Tff3^−/−^* female mice had lower glucose levels compared to WT females 15 and 30 min after the initial injection of the first glucose dose (Figure S2b). After 6 weeks on a high-fat diet, these differences were no longer detectable (Figure S3). The results indicate differences in the response to glucose in *Tff3^−/−^* animals compared to WT animals, without the additional stress of a high-fat diet, whereby a 6-week high-fat diet leads to a reduction in the mentioned differences. These results could suggest a possible adaptive mechanism in *Tff3^−/−^* animals at the onset of nutritional stress. In addition, the outcome of the tests was sex-dependent, i.e., the results were different for *Tff3^−/−^* males and *Tff3^−/−^* females compared to the corresponding WT control. A comparison with the results of the metabolic tests carried out on a model using a long-term high-fat diet shows that factors such as the duration of treatment and the age of the animal significantly influence the outcome of the tests [15]. For example, *Tff3^−/−^* males show no differences in glucose tolerance compared to WT males after 6 weeks on a high-fat diet (Figure S3a), whereas *Tff3^−/−^* males show better glucose tolerance after 17 weeks on a high-fat diet, and after 36 weeks this difference disappears [15].

Regarding the results of insulin sensitivity tests, there are no differences in either males or females before exposure to a high-fat diet (Figure S4), but after 7 weeks of a high-fat diet, *Tff3^−/−^* males have significantly better insulin tolerance at almost all time points compared to WT males (Figure S5a). The results indicate the possible protective role of Tff3 deficiency in the context of insulin sensitivity after 7 weeks of high-fat-diet treatment. Compared to WT females, *Tff3^−/−^* females do not show differences in insulin sensitivity, either on a standard or a high-fat diet (Figures S4b and S5b). A comparison of the results of insulin tolerance tests with the long-term fatty diet model once again indicates the difference in effect depending on the length of the treatment and the age of the animals [15].

Metabolic studies were previously performed on *Tff3^−/−^* mice of mixed lineage (C57BL6/J129SV) and showed that Tff3-deficient mice exhibit better glucose and insulin tolerance compared to the corresponding wild-type controls [10]. In this case, the results suggest the protective effect of Tff3 protein deficiency, and the animals used were 12-week-old males fed a standard diet. In our study, *Tff3^−/−^* C57BL/6N males fed a standard diet showed impaired glucose tolerance compared to wild-type animals, but only at the 30 min time point (Figure S2a), whereas there was no difference in insulin sensitivity (Figure S4a). The different observed outcomes of metabolic tests could be due to the age of the animals or the different genetic background of the strain. *Tff3^−/−^* mice of mixed genetic origin were developed on the C57BL6/J background, which we know has mutations relevant to metabolic processes [25]. Furthermore, in addition to the age of the animals and the type of strain, various factors such as sex, the type and duration of treatment, and the protocol of the test itself can drastically influence the outcomes of metabolic tests. For this reason, it is difficult to compare the studies and the need for the standardisation of the tests and a clear indication of the protocol and the animal model used in the study is emphasised.

From the results obtained in our study, it appears that Tff3 plays a role in glucose homeostasis and insulin sensitivity even without the additional stress of a high-fat diet. *Tff3^−/−^* males have better insulin sensitivity than WT controls after 7 weeks on a high-fat diet, suggesting the protective effect of Tff3 deficiency on insulin metabolism.

### 4.2. Liver Lipid Analysis

The role of Tff3 in animal models of MAFLD has already been observed. Precisely, mouse models of obesity and diabetes (ob/ob, db/db, DIO-C57BL6J) display a fatty liver phenotype characterised by the increased accumulation of lipid droplets and hepatocyte damage, and the downregulation of Tff3 expression in the liver is observed in these models [3,4,5]. The restoration of Tff3 expression in the mentioned models leads to an improvement in the phenotype of fatty liver, i.e., a reduction in lipid droplets and hepatocyte damage, whereas the suppression of Tff3 expression in the liver has the opposite effect, i.e., it leads to increased lipid accumulation [5]. Considering the results of this study, we hypothesised that Tff3^−/−^ mice might exhibit increased lipid accumulation in the liver. However, after 9 weeks of HFD, male and female Tff3^−/−^ mice exhibited a smaller size of lipid droplet in the liver than WT animals (Figure 2), although total fat content per /g liver was not affected (Table 1). Tff3-deficient mice in the long-term high-fat-diet model had lower fat accumulation in the liver compared to WT controls [15]. Thus, our research shows that congenic *Tff3* deficiency in the short term does not affect the total level of fat in liver (but rather size of lipid vesicles), while prolonged exposure reduces the level of fat accumulation in liver.

Since an imbalance in fatty acid metabolism and the content of fatty acids play important roles in the development of MAFLD [26], we further analysed the composition of specific fatty acids in the liver of Wt- and Tff3-deficient mice (Table 1). In general, research suggests that certain types of fatty acids, such as saturated fatty acids (SFAs), contribute to the development of more severe forms of disease such as steatohepatitis and cirrhosis and accelerate their progression, while omega-3 polyunsaturated fatty acids (n-3/ PUFA) have anti-inflammatory properties and may help to reduce the accumulation of fat in the liver, thus having a protective effect [27,28,29,30]. The results of the fatty acid analysis show the significantly reduced content of arachidonic fatty acid (C 20:0) in the liver of *Tff3^−/−^* male after 9 weeks of a high-fat diet compared to the WT male control (Table 1). Arachidonic fatty acid belongs to the group of SFAs, and studies indicate the toxic effect of excessive SFA intake on liver homeostasis [30]. The mechanisms of action behind this are not yet fully understood, but include the activation of endoplasmic reticulum stress, mitochondrial dysfunction, and the accumulation of reactive oxygen species, leading to liver damage [30,31,32]. Apart from arachidonic acid, the results show no other differences in fatty acid levels in the liver of *Tff3^−/−^* males compared to WT males. Compared to WT females, *Tff3^−/−^* female mice had lower levels of gadoleic acid (C20:1, n-9) and higher levels of several PUFAs (linoleic acid (C 18:2, n-6), linolenic acid (C 18:3, n-6), eicosapentaenoic acid (C 20:5, n-3), and docosapentaenoic acid (C 22:5, n-3)), contributing to the higher total omega-3 PUFA content in the liver.

Studies in animal models as well as some clinical studies show the potential of omega-3 PUFA supplementation in the treatment of MAFLD [33,34,35]. An increased intake of omega-3 PUFA leads to a reduction in hepatic steatosis, an improvement in insulin sensitivity, and a reduction in inflammatory markers [20,36]. Thus, the increased level of omega-3 PUFA in the liver of *Tff3^−/−^* females compared to WT controls suggests a possible protective effect of Tff3 deficiency due to short-term exposure to a high-fat diet. This effect is related to fatty acid composition in the liver. Research on this protective role is usually based on a combination of eicosapentaenoic acid (EPA) and docosahexaenoic acid (DHA) intake. EPA was also statistically significantly increased in the livers of *Tff3^−/−^* females compared to WT controls (Table 1), while significantly reduced levels were found in patients diagnosed with MAFLD and more severe forms of the disease such as non-alcoholic steatohepatitis compared to healthy controls [37,38,39]. Another indicator used in the study of MAFLD is the ratio of omega-6 to omega-3 polyunsaturated fatty acids (n-6/n-3 PUFA). A typical Western diet is characterised by an increased intake of omega-6 fatty acids and a reduced intake of omega-3 fatty acids, and this imbalance has been shown to contribute to the MAFLD phenotype [40,41,42]. Increased levels of n-6/n-3 PUFA correlate with increased inflammation and fat accumulation in the liver, and diets aimed at reducing this ratio have been recognised as potential means of preventing and/or treating diseases such as MAFLD. *Tff3^−/−^* females had a statistically significantly lower ratio of n-6/n-3 PUFA fatty acids in the liver than WT females (Table 1).

To summarise, the differences found in blood biochemistry (males had lower levels of triglicerides and females had significantly lower levels of n6/n3 PUFA), which have a positive effect on liver inflammation, show that the Tff3 deficiency effect on the lipid metabolism is strongly sex-dependent.

### 4.3. Expression of Genes Related to Relevant Pathophysiological Pathways

Tff3 gene expression was analysed using a qPCR method in the liver of WT animals of both sexes, employing both standard and high-fat-diet treatment. As mentioned previously, it has been found that Tff3 liver expression is reduced in various mouse models of obesity and diabetes (genetic and dietary) [3,4,5] compared to healthy controls. In addition, Tff3 gene expression was statistically significantly reduced in the liver of C57BL6/N males exposed to long-term treatment with a high-fat diet (36 weeks) compared to controls on a standard diet [15]. This effect was not detected in this study (Figure S6c,d), suggesting the possibility that 9 weeks of a high-fat diet is not sufficient to observe a statistically significant difference in Tff3 downregulation in the liver of C57BL6/N mice. Regarding the influence of sex on the expression of the Tff3 gene in the liver of C57BL6/N mice, we confirmed that the expression of Tff3 is extremely low in the liver of females compared to males (Figure S6a,b). The same result was observed in animals fed a standard diet (Figure S6a), as well as in animals exposed to a high-fat diet (Figure S6b). The same phenomenon was previously observed in the livers of C57BL/6JOIaHsd females [43]. Our findings align with previous observations in C57BL6/N mice [15], although alternative explanations should be considered.

Given the observed differences in liver lipid accumulation between *Tff3^−/−^* and WT animals exposed to a short-term high-fat-diet treatment, we investigated the effects of Tff3 deficiency on genes involved in the development of metabolic disorders, with a focus on MAFLD (Figure 3). The deregulation of hepatic lipid metabolism, leading to steatosis, may be due to several mechanisms, including β-fatty acid oxidation, increased de novo lipogenesis, and decreased triglyceride excretion [26]. The imbalance of the mentioned processes promotes the development of metabolic disorders. Using qPCR analysis, we determined the gene expression of various markers of lipid metabolism in the livers of WT and *Tff3^−/−^* animals of both sexes (Figure 3a,b). Since it was previously observed that Tff3 can bind to the transcription factor PPARα and thereby increase markers of β-oxidation of fatty acids in the liver [5], the expression of the marker genes *Pparα*, *Cpt1α,* and *Hgmcs2* was analysed. However, significant differences were not detected in *Tff3^−/−^* animals compared to WT controls (Figure 3a,b). This is consistent with previous findings from our research group, where no changes in the gene expression of *Pparα* and *β-oxidation* markers in the liver were detected for *Tff3^−/−^* males and WT males exposed to a long-term high-fat diet [15]. However, the level of the transcription factor *Pparγ* was reduced in the liver of *Tff3^−/−^* males compared to WT males after a long-term high-fat diet [44]. Previously, reduced levels of Pparγ protein were also observed in the liver of *Tff3^−/−^* mice (C57BL6/J/129sv) on a standard diet [15], but qPCR analysis in this study showed no statistically significant differences in the levels of the Pparγ gene (as well as protein level) and its downstream genes *Cyp21* and *Scd1* in the short-term diet model (Figure 3a,b). Furthermore, markers (*Srebpc1, Chrebp, Fasn, Lxr, Elov*) were analysed to investigate the hypothesis that the observed reduced lipid accumulation in the liver of *Tff3^−/−^* animals could be due to a decreased rate of de novo lipogenesis (Figure 3a,b). However, there were no statistically significant differences in the gene expression of de novo lipogenesis markers between WT and Tff3^−/−^ animals. Also, no difference was found in the markers of lipid droplet formation (*Dgat1*, *Fitm2*), lipolysis (*Cgl58*) and gluconeogenesis (*GyK*) (Figure 3a,b).

The only detectable statistically significant difference was the increased level of *Irs2* in the liver of *Tff3^−/−^* females compared to WT females (Figure 3b). Irs2 is one of the key mediators of insulin signalling that regulates the expression of various processes that can lead to increased lipid accumulation in the liver [45]. Insulin binds to the insulin receptor, which enhances the phosphorylation of Irs1 and Irs2 substrates in hepatocytes. It has been shown that *Irs2^−/−^* mice have impaired pancreatic β-cell function and consequently develop systemic insulin resistance and hyperglycaemia [46]. However, mice where Irs2 is silenced, specifically in the liver, show selective insulin resistance, in which insulin no longer suppresses the process of gluconeogenesis but continues to activate lipogenesis [47]. In addition, they show signs of hepatic steatosis and have impaired glucose tolerance. Furthermore, the expression of Irs2 was found to be significantly reduced in the livers of patients with type 2 diabetes [48]. Increased Irs2 gene expression in the liver of *Tff3^−/−^* females compared to WT females after a short-term high-fat diet (Figure 3b), as well as the protective fatty liver phenotype observed (Figure 2 and Table 1), is in line with the deleterious effect of Irs2 gene silencing on the fatty liver phenotype, which is reported in the studies mentioned. This effect has been shown to be due in part to increased lipogenesis [47]; however, the analysis of de novo lipogenesis markers shows no changes in the liver of *Tff3^−/−^* females compared to WT females (Figure 3b). Furthermore, there are also no differences in insulin sensitivity in metabolic assays (Figures S4b and S5b), and so it is possible that the increased Irs2 gene expression detected is due to the regulation of a different mechanism, as Irs2 plays a role in a variety of processes including cell growth, development, and survival.

The pathogenesis of MAFLD is a complex and multifactorial process, and an increased inflammatory state is one of the driving forces behind progression to more severe forms of the disease, such as non-alcoholic steatohepatitis with increasing liver damage [26]. Considering that the Tff3 protein is involved in regulating the immune response of the GI tract [49,50], we monitored the gene expression of relevant inflammatory markers in the livers of *Tff3^−/−^* and WT mice exposed to a short-term high-fat-diet treatment, hypothesising that there may be differences in the immune response between *Tff3^−/−^* and WT animals (Figure 3c,d). In the liver of *Tff3^−/−^* males, the levels of cytokines *Il1α* and *Cxcr7* were reduced compared to WT males (Figure 3c), while in *Tff3^−/−^* females there were no statistically significant differences in the analysed genes compared to WT females (Figure 3d). Il1α, together with Il1β, belongs to the interleukin-1 family, which has been much more studied in the context of metabolic diseases, and its inhibition is one of the pharmacological strategies being tested as a treatment for type 2 diabetes [51]. However, recent evidence also points to the potential role of Il1α in the same processes. Namely, it was discovered that *Il1α^−/−^* mice exhibit lower serum triglyceride and cholesterol levels and the complete inhibition of triglyceride accumulation in the liver after treatment with a high-fat diet [52]. This has been shown to be due to the inhibition of key enzymes of de novo lipogenesis in the liver. *Il1α* is downregulated in the liver of Tff3-deficient mice, and as these animals also exhibit reduced serum triglyceride levels and hepatic fat accumulation, this study may provide a possible link. However, as mentioned above, the analysed gene levels of markers of de novo lipogenesis in the liver show no differences between Tff3^−/−^ males and WT controls (Figure 3a). In addition to *Il1α*, the level of *Cxcr7* was also decreased. Cxcr7, found in the liver, may be involved in regulating the migration of immune cells [53], and the interaction between Tff3 and Cxcr7 has been previously demonstrated, albeit in the epithelial cells of the conjunctiva of the eye [54].

In addition to inflammation, endoplasmic reticulum stress and oxidative stress are important features of the pathophysiology of metabolic disorders [55]. An overload of saturated fat, i.e., nutritional excess, can cause stress that activates the unfolded protein response (UPR) with the aim of restoring the homeostasis of the endoplasmic reticulum function. UPR activity is mediated by three membrane proteins—inositol-requiring protein 1 alpha (IRE1 α), protein kinase RNA-like ER kinase (PERK), and activating transcription factor 6 (ATF6)—which then regulate the expression of various genes involved in various cellular processes (apoptosis, inflammation, lipid metabolism, autophagy, etc.) via a further cascade. Tff peptides, including Tff3, have previously been linked to ER stress and oxidative stress [56,57,58,59]. Therefore, we monitored UPR activation and oxidative stress markers by qPCR analysis. The only statistically significant difference observed was the increased level of *Atf4* in *Tff3^−/−^* females compared to WT females (Figure 3e,f). Atf4 is a transcription factor that belongs to the cAMP-responsive element-binding (CREB) protein family, and in the context of its role in the UPR, it plays a role in PERK/ATF4/CHOP signalling, which is crucial for the initiation of cell apoptosis in the case of sustained ER damage [60]. Since no significant difference was observed in the level of the Chop gene (Figure 3f), we assume that the increased level of Atf4 in *Tff3^−/−^* females is not related to apoptotic function. Several studies have pointed to the role of the Atf4 protein in the regulation of lipid metabolism. Atf4 deficiency leads to the inhibition of fat accumulation in the liver of mice exposed to various treatments (high-carbohydrate diet [61] and fructose [62]) and to a generally favourable metabolic state under standard diet conditions (protection against age-related weight gain, as week as better glucose tolerance and insulin sensitivity) [63]. The exact mechanisms are not yet fully understood, but it has been shown that the absence of Atf4 leads to a decrease in the expression of lipogenesis markers in the liver [62]. Thus, *Atf4^−/−^* mice do not develop the MAFLD phenotype when exposed to different treatments. However, in *Tff3^−/−^* females, Atf4 levels in the liver are increased and lipid content is reduced compared to WT controls. In addition, no significant differences in lipogenesis markers are detected (Figure 3b), and so increased levels of the Atf4 gene in *Tff3^−/−^* females could either be related to other functions/processes, affected by this transcription factor, or due to a different regulatory mechanism. In this case, the increased level had a favourable effect on the fatty liver phenotype, but elucidating the exact reasons for this requires further research.

qPCR analysis revealed differential gene expression in relevant pathways, but functional validation is needed. After short-term high-fat-diet exposure, *Tff3^−/−^* males have reduced levels of *Il1α* and *Cxcr7*, while *Tff3^−/−^* females have increased levels of *Irs2* and *Atf4* genes compared to WT controls (Figure 3). In general, markers involved in lipid metabolism, inflammation, endoplasmic reticulum stress, and oxidative stress did not reveal major differences that would explain the cause of the observed protective phenotype. However, the genes that were significantly altered provided valuable direction for the further investigation of the possible molecular mechanisms of Tff3 action in the liver.

In addition, we wanted to investigate whether these differences were a consequence of the Tff3 deficiency associated with the response to stress exposure to foods rich in saturated fat, or whether they were also present without treatment, i.e., in animals on a standard diet (Figure S7). There were no statistically significant changes in the levels of the markers studied, suggesting that the differences mentioned occur when treated with a high-fat diet.

### 4.4. LC MS/MS Analysis of Liver in WT and Tff3^−/−^ Mice Exposed to Short Term High Fat Treatment

In addition to gene expression analysis of various relevant markers, LC/MS MS analysis was performed using a Q-Exactive Plus mass spectrometer to detect changes in protein expression in the liver of *Tff3^−/−^* and WT animals of both sexes exposed to short-term high-fat-diet treatment. Although differences were detected in the previously mentioned analyses and tests, e.g., differences in body weight (Figure 1b), IPITT (Figure S5a), serum triglyceride levels (Figure S1), Oil Red O staining and fatty acid composition (Figure 2 and Table 1), and the level of Il1α and Cxcr7 gene expression (Figure 3c), no statistically significant changes were detected in the protein levels in liver of *Tff3^−/−^* males compared to WT males after he application of the statistical FDR tool. Although there is no statistical significance, the possibility that there are biologically significant altered proteins in the liver of *Tff3^−/−^* males compared to WT males is not excluded. It is merely not detected using this method.

However, the results show that *Tff3^−/−^* females have altered levels of a total of 28 proteins compared to WT females (Table 2 and Figure 4). Among them are ribosomal proteins (Rpl17, Rpl19, Rpl26, Rpl28, Rsp13), which are upregulated in the liver of *Tff3^−/−^* females (Table 2 and Figure 4). The increased expression of ribosomal proteins suggests that ribosome biosynthesis may be increased in the livers of *Tff3^−/−^* females compared to WT females. Ribosome biosynthesis is one of the most energy-intensive processes required for cell growth, proliferation, and differentiation and is therefore essential for the maintenance of cell homeostasis and normal development [64]. The speed and rate of ribosome synthesis depends on several factors. Among other effects, it has been shown that a high-fat diet leads to a decrease in rRNA transcription in the liver of mice, resulting in decreased oxidation and increased synthesis of lipids and consequently increased fat accumulation in the liver of these mice [65]. In view of this, one of the mechanisms leading to reduced fat accumulation in the liver of Tff3-deficient mice could be mediated by increased ribosome biosynthesis. Using the software tool STRING (https://string-db.org/ accessed on 6 April 2025), the identified proteins were analysed and the interaction of the mentioned ribosomal proteins with the Ybx1 protein was detected. The Ybx1 protein was upregulated in the liver of *Tff3^−/−^* females compared to WT females, which was confirmed by the Western blot method (Figure 5). Ybx1 is a multifunctional protein that can bind to nucleic acids (DNA/RNA). It has a variety of functions, such as regulating the transcription and translation of various genes involved in various processes (proliferation, survival, migration), mRNA splicing, DNA repair, etc. [66]. STRING analysis showed the interaction of Ybx1 with the Hnrnpd protein (Figure 4). It has been shown that Ybx1 can bind to Hnrnpd, and their interaction results in endonuclease activity, i.e., the degradation of mRNA, which may be an important mechanism in the control of gene expression [67,68]. In addition to the link between Ybx1 and Hnrnpd, STRING analysis indicated the interaction of Hnrnpd with the Sfpq protein, for which proteomic analysis shows upregulation in the liver of *Tff3^−/−^* females compared to WT variants. The analysis of the expression of genes involved in RNA processing in liver samples from patients with obesity and insulin resistance showed that Sfpq and Hnrnpd were among the most altered factors compared to healthy controls [69]. Both factors were significantly reduced in the livers of patients, whereas these proteins were overexpressed in the livers of *Tff3^−/−^* females.

Furthermore, STRING analysis identified a ’network’ comprising proteins from the histone family (Hist1h2bp, H2afv, H2afx) and the protein Anp32a (Figure 4). These proteins were found to be upregulated in the liver of *Tff3^−/−^* females compared to WT females (Table 2). We also confirmed the increased level of Anp32 protein using the Western blot method (Figure 5). Anp32a is a protein with a variety of functions, such as the regulation of transcription, the maintenance of mRNA stability, DNA repair, and the regulation of apoptosis. Additionally, in the context of interaction with histones, it is part of the INHAT (Inhibitor of Histone Acetyltransferases) complex, which, as the name implies, is involved in the inhibition of histone acetylation [70]. Histone acetylation is one of the epigenetic modifications that regulates gene expression by modulating the chromatin structure [71]. Furthermore, a correlation has been established between the inhibition of histone acetylation and the regulation of genes involved in lipid metabolism. For example, treatment with an inhibitor of p300 histone acetyltransferase significantly reduced the number of lipid droplets and the gene expression of lipogenesis markers [72]. In addition, Anp32a deficiency, which leads to reduced histone H3 acetylation, is associated with changes in gene expression during lipid metabolism [73]. The changes observed or the low number of altered proteins could be due to the protein isolation technique (novel column concept with minimal use of detergents), but they certainly show that there are sex-specific differences in the transcription machinery that we cannot explain at the moment.

Among all physiological changes noticed in the liver, the most relevant finding is that, although total liver lipid content is similar in both strains, the size of lipid droplets is smaller in Tff3-deficient mice that seem to have lower number of large lipid droplets (Figure 2). Lipid droplets (LDs) are organelles of cellular lipid storage with fundamental roles in energy metabolism and cell membrane homeostasis [74]. They are dynamic organelles that store neutral lipids, such as triacylglycerols (TGs) and cholesterol esters (CE), which can be used to generate metabolic energy or cell membranes. Long regarded merely as inert fat reservoirs, they are now emerging as major regulators of cellular metabolism. They act as hubs that coordinate the pathways of lipid uptake, distribution, storage, and use in the cell. Specific proteins, including many important lipid metabolism enzymes (e.g., TG synthesis and degradation enzymes), bind to LD surfaces that are related to cellular function damage. Recent studies have revealed that they are also essential components of the cellular stress response [74]. There has been an explosion of research into the biology of LDs, in part due to their relevance in diseases of lipid storage, such as atherosclerosis, obesity, type 2 diabetes, and hepatic steatosis [75]. Recently, it was shown that liver steatosis progression in vivo is related to changes in protein localization, organelle organisation, and protein phosphorylation [76]. Several organelle contact site proteins are targeted to lipid droplets (LDs) in steatotic liver, tethering the organelles that orchestrate lipid metabolism. Proteins of the secretory pathway are dramatically redistributed, including the mis-localization of the COPI complex and the sequestration of the Golgi apparatus at LDs. This correlates with reduced hepatic protein secretion. In this way, nutrient overload leads to organellar reorganisation and cellular dysfunction.

## 5. Conclusions

Monitoring the effect of Tff3 protein deficiency in models of nutritional overload, we discovered the protective effect of Tff3 deficiency on lipid accumulation in the liver in models of mice after both short- and long-term HFD exposure, which is inconsistent with some other mouse models. The difference may be due to several factors already mentioned, such as the genetic background of the strain, the duration and type of treatment, and the age and sex of the animals, which affect metabolic properties and thus emphasise the complexity of the regulation of lipid metabolism. However, the main difference between the studies mentioned above, in which Tff3 was shown to have a positive effect on the metabolic phenotype (in this case, it is a reduction in fat accumulation in the animal), and the results of our research group, in which Tff3 deficiency led to a protective effect, is the experimental model. It appears that the forced adenoviral overexpression of Tff3 has the same effect in mice suffering from steatosis. Our mouse model has no additional mutation, and we mimic the physiological approach of feeding mice a fat-saturated diet, which covers all possible aspects of Tffs function, including aspects of its role at the intestinal surface and in the bloodstream. Tff3^−/−^/C57Bl6N mice were protected from lipid accumulation in the liver after 9 weeks of HFD. Biochemical analysis of sera only showed reduction in triglyceride levels in Tff3-co males, but not in females. The accumulation of lipids in the liver was not affected at the level of total lipid content, but rather at the level of distribution (size of vesicles). Tff3^−/−^ males had statistically a significantly lower body weight, better insulin tolerance, reduced serum triglyceride levels, lower liver lipid discoloration, lower saturated arachidonic acid content, and lower expression of the Il1α and Cxcr7 genes compared to WT males, while we did not detect relevant changes in the liver proteome. Tff3^−/−^ females also had lower body weight, lower liver lipid staining at the same fat content, a reduced ratio of omega-6 to omega-3 PUFAs, and increased expression of Irs2 and Atf4 genes compared to WT females, while proteomic analysis revealed altered levels of proteins involved in ribosome biosynthesis and the inhibition of acetylation. The presence of Tff3 protein on intestinal surface, in epithelial gall balder mucosa (Figure S8), and in blood circulation confirms its ability to act at distant sites, which is a well-known phenomenon. In view of this, it should be considered that the effects of Tff3 deficiency in other organs or interactions between different organ systems (especially digestion/absorption) are part of the cause of the protective phenotype observed. Different phenotypes in different mouse models (e.g., multigenic disease models or mice with different genetic backgrounds) and different effects on induced genetic manipulations (e.g., local expression) need to be considered in the context of physiological localization and function. In this context, our Tff3-deficient mouse models are a good starting point to uncover the actual physiological role of Tff3 in complex metabolic processes.

## Figures and Tables

**Table 1:** Fatty acid content in liver of WT and Tff3^−/−^ animals of both sexes after short-term HFD exposure.

Main Fatty Acid Group (g of Fatty Acids/100 g of Total Fatty Acids)	HFD WT ♂	HFD Tff3^−/−^ ♂	HFD WT ♀	HFD Tff3^−/−^ ♀
C 14:0	0.43	0.41	0.42	0.44
C 16:0	25.38	24.58	24.76	25.20
C 16:1	4.06	3.85	3.07	2.88
C 18:0	8.41	9.45	8.76	10.21
C 18:1	32.76	30.45	37.40	31.80
C 18:2, n-6	10.19	11.27	9.27 ^§^	10.47
C 18:3, n-6	0.33	0.37	0.32 ^§^	0.49
C 18:3, n-3	0.14	0.17	0.12	0.16
C 20:0	0.34 ^‡,^*	0.27 ^†^	0.03	0.04
C 20:1, n-9	0.56	0.46 ^†^	0.42 ^§^	0.25
C 20:3, n-6	0.91 *	0.97 ^†^	0.60	0.62
C 20:4, n-6	10.00	10.73	8.69	9.81
C 20:5, n-3	0.12 *	0.14	0.07 ^§^	0.13
C 22:4, n-6	0.33	0.32	0.32	0.37
C 22:5, n-6	0.49	0.49 ^†^	0.57	0.62
C 22:5, n-3	0.31 *	0.33	0.16 ^§^	0.28
C 22:6, n-3	4.39	4.78	4.25	5.37
∑ SFA ^1^	35.00	35.19	34.35	36.34
∑ MUFA ^2^	37.47	34.81	40.90	34.96
∑ PUFA ^3^	27.19	29.68	24.44	28.33
n-6 PUFA	22.14	24.08	19.73	22.13
n-3 PUFA	4.98	5.46	4.63 ^§^	5.99
n-6/n-3 PUFA ^4^	4.59:1	4.42:1 ^†^	4.29:1 ^§^	3.71:1
Total fat content (g/100 g liver)	6.50 ± 2.36	5.22 ± 0.24	7.41 ± 1.11	5.96 ± 1.58

Results are presented as mean and mean ± SD (for fat content) and were analysed using general linear model (GLM) procedures of the SAS/STAT module (SAS Institute Inc., Cary, NC, USA), with the differences determined by a Tukey–Kramer multiple comparison test. This took the genotype into consideration as the main effect, albeit separately for male and female mice. Statistical significance was as follows: *p* ≤ 0.05. * WT ♂ vs. WT ♀ (sex-related diff.). ^†^ Tff3^−/−^o ♂ vs. Tff3^−/−^ ♀ (sex-related diff.). ^‡^ WT♂ vs. Tff3^−/−^ ♂ (gene-related diff.). ^§^ WT ♀ vs. Tff3^−/−^ ♀ (gene-related diff.). ^1^ saturated fatty acids. ^2^ monounsaturated fatty acids. ^3^ polyunsaturated fatty acids. ^4^ ratio of omega-6 to omega-3 polyunsaturated fatty acids.

**Table 2:** Results of LC MS/MS analysis—proteins with significantly changed expression in the liver of Tff3^−/−^ females compared to WT females exposed to a high-fat diet for 9 weeks.

Protein Name (Short in STRING)	Fold Change	FDR	*p* Value
ribosomal protein L19 (Rpl19)	1.491	0.046	0.002
histone H2B type 1-P isoform 1 (Hist1h2bp)	1.341	0.046	0.002
nucleolar and coiled-body phosphoprotein 1 (Nolc1)	1.334	0.046	0.002
histone H2afv (H2afv)	1.331	0.046	0.002
60S ribosomal protein L28 isoform X1 (Rpl28)	1.327	0.046	0.002
splicing factor proline/glutamine-rich (polypyrimidine tract binding protein-associated) (Sfpq)	1.314	0.046	0.002
nuclease-sensitive element-binding protein 1 isoform X1 (Ybx1)	1.305	0.046	0.002
signal recognition particle subunit (Srp27)	1.264	0.046	0.002
ubiquinol-cytochrome c reductase, complex III subunit VII (Uqcrq)	1.264	0.046	0.002
ribosomal proteinL26 (Rpl26)	1.261	0.046	0.002
mannose-binding protein C precursor (Mbl2)	1.247	0.046	0.002
histone H2A.X (H2afx)	1.215	0.046	0.002
ribosomal protein (Rpl17)	1.186	0.046	0.002
acidic nuclear phosphoprotein pp32 (Anp32a)	1.182	0.046	0.002
heterogeneous nuclear ribonucleoprotein AU-rich element RNA-binding protein 1 (Hnrnpd)	1.151	0.046	0.002
ribosomal protein S13 (Rsp13)	1.117	0.046	0.002
vesicle-associated membrane protein, associated protein B and C (Vapb)	1.107	0.046	0.002
enoyl coenzyme A hydratase 1, peroxisomal (Ech1)	1.082	0.046	0.002
protein disulfide-isomerase A4 isoform 1 precursor (Pdia4)	1.073	0.046	0.002
peptidylprolyl isomerase B (Ppib)	1.073	0.046	0.002
phosphatidylinositol binding clathrin assembly protein (Picalm)	1.055	0.046	0.002
sec14-like protein 2 (Sec14l2)	0.920	0.046	0.002
dipeptidyl peptidase 4 isoform 1 (Dpp4)	0.914	0.046	0.002
2-hydroxyacyl-CoA lyase 1 (Hacl1)	0.911	0.046	0.002
fumarylacetoacetase hydrolase (Fah)	0.895	0.046	0.002
gtp cyclohydrolase I feedback regulator (Gchfr)	0.820	0.046	0.002
aldehyde dehydrogenase, cytosolic 1 (Aldh1a7)	0.808	0.046	0.002
3-hydroxy-3-methylglutaryl-Coenzyme A synthase 1 (Hmgcs1)	0.792	0.046	0.002

Analysis was performed using SEQUEST algorithms and the Proteome Discoverer software programme. For statistical analysis, a limit of 5% FDR was used, calculated using the Percolator algorithm, and the unpaired *t*-test was used to calculate the value; then the Benjamini–Hochber correction was applied.

## Data Availability

The original contributions presented in this study are included in the article/Supplementary Materials. Further inquiries can be directed to the corresponding author(s).

## Supplementary Materials

The following supporting information can be downloaded at https://www.mdpi.com/article/10.3390/biomedicines13051024/s1. Table S1. Oligonucleotides used for qPCR analysis. Figure S1: Effect of Tff3 deficiency on serum biochemical parameters after 9 weeks of high-fat diet. Figure S2. Intraperitoneal glucose tolerance test (IPGTT) performed on 9-week-old mice exposed to a standard diet. Figure S3. Intraperitoneal glucose tolerance test (IPGTT) performed on 17-week-old mice exposed to a high-fat diet for 6 weeks. Figure S4. Intraperitoneal insulin tolerance test (IPITT) performed on 9-week-old mice exposed to standard diet. Figure S5: Intraperitoneal insulin tolerance test (IPITT) performed on 18-week-old mice exposed to a high-fat diet for 7 weeks. Figure S6. Tff3 gene expression in the liver of C57BL6/N mice fed with standard and high-fat-diet. Figure S7. Gene expression of markers Cxcr7, Il1α, Irs2 and Atf4 in the liver of WT and Tff3^−/−^ mice exposed to SD. Figure S8. (A) Localisation of Tff3 protein in (A) colon, liver/ gall bladder of Wt- and Tff3-deficient mice. (B) No prominent presence of TFf3 in the liver of mice on SD and HFD; (C) expression of Tff3 in gall balder is reduced upon HFD exposure.
