# Supplementary material for: The Effect of Tff3 Deficiency on the Liver of Mice Exposed to a High-Fat Diet
Source: Biomedicines. 2025 Apr 23;13(5):1024. doi: 10.3390/biomedicines13051024 (PMC12108639; doi:10.3390/biomedicines13051024)
Supplement: Supplementary file 1 [file biomedicines-13-01024-s001.zip › biomedicines-3528488-supplementary.pdf]

Table S1. Oligonucleotides used for qPCR analysis

Gene name	Gene Symbol	Accession No.	Primer Sequence Forward (5′-3′) Reverse (5′-3′)	**Optimized qPCR Conditions (Annealing Temperature/MgCl concentration)**
		Lipid metabolism markers		
abhydrolase domain containing 5	*Cgl58*	NM_026179.2	ATGCTGTGGAATGAG-GACATAG CATAGTGAGTGGCTGGTGAAA	59°C; 2,5 mM
carbohydrate response element binding protein	*Chreb*	NM_021455.5	CAGCTGCGGGATGAAATAGA CAAAGCGCTGATGTGTGATG	61°C; 2,5 mM
carnitine palmitoyltransferase I	*Cpt1α*	NM_013495.2	TCGAAACCCAGTGCCTTAAC AAGCAGCACCCTCACATATC	58°C 2,5 mM
cytochrome P450, family 21, subfamily a, polypeptide	*Cyp21*	NM_009995.2	CTGGGTCGGAGCTTCATTT GTCTTGACTCTCTTCCCTTGAC	59°C; 3,5 mM
diacylglycerol O-acyltransferase 1	*Dgat1*	NM_010046.3	CCAACCATCTGATCTGGCTTAT GACTCAGCATTCCACCAATCT	65°C; 3 mM
elongation of long-chain fatty acids family member 6	*Elov6*	NM_130450.2	CCGAACTAGGTGACACGATATT GTAGGAGTACCAGGAGTACAG	65°C; 3 mM
fumarylacetoacetate hydrolase	*Fah*	NM_010176.4	TAACTCAAAGCCTCCTGTG-TATG GATTGGCTCTCCGAATCTGTT	62°C; 2,5 mM
fatty acid synthase	*Fasn*	NM_007988.3	CCTGACCAAGGTGCTGTTAT CATCAAGAAGTGCTGGGATCT	65°C; 2,5 mM

fat storage-inducing transmembrane protein 2	*Fitm2*	NM_173397.4	GACAGGAGGACAATGGCTAAT CCACACCAAAGGTACCTAG-TAAG	56°C; 2,5 mM
glycerol kinase	*Glyk*	BC003767.1	GCACTAGAAGCTGTTTGTTTCC GCTGGTCATTCCTCCATCTAC	58°C; 2,5 mM
hydroxymethylglutaryl-CoA synthase	*Hmgcs2*	NM_008256.4	CCTGTGAAGAGGGA-GATGAAAG GCCCACAGTCTGA-GAATAAGC	64°C; 3 mM
insulin receptor substrate 1	*Irs1*	NM_010570.4	GTCAGGGACACTCTTGACTAAC TGCCAAGGAAAGACAGGA-TAAA	61°C; 2,5 mM
insulin receptor substrate 2	*Irs2*	NM_001081212.2	CTGCTGCTCACTTTCCTATCA CCTGCCTCTTGGTTCCTTATC	61°C; 2,5 mM
nuclear receptor subfamily 1	*Lxra*	NM_013839.4	GGGCCTAATGAAGCTAA-GAGTC GCAGGGAGAAGCAG-TAATGTAG	65°C; 2,5 mM
peroxisome proliferator activated receptor alpha VI	*Pparα*	NM_011144.6	GCTCGTACAGGTCATCAA-GAAG CTGCCATCTCAGGAAAGATCAG	59°C 2,5 mM
peroxisome proliferator activated receptor gamma	*Pparγ*	NM_001127330.2	GCCTAAGTTTGAGTTTGCTGTG GCGGTCTCCACTGAGAATAATG	59°C 2,5 mM
stearoyl-CoA desaturase 1	*Scd1*	NM_009127.4	GGCAGTTCTGAGGTGATTAGAG GTCTCTGGGAAGAGCAATGTAG	60°C; 2,5 mM
sterol regulatory element binding transcription factor 1	*Srebpf1*	NM_011480.4	AGCCCTCCACCAGGTAATAA GGGTTCCCAGTCTACTCACTAA	61°C; 2,5 mM
Inflammation markers				
chemokine (C-C motif) receptor 2	*Ccr2*	NM_009915.2	GGTCTGGTTGGGTTGTAAA GTCTTTGAGGCTTGTTGCTATG	59°C; 3 mM
mouse CD68 antigen	*Cd68*	NM_001291058.1	CTCTTGCTGCCTCTCATCATT CTGGTAGGTTGATTGTCGTCTG	58 °C; 2,5 mM

C-X-C motif chemokine ligand 1	*Cxcl1*	NM_008176.3	GTGTCAACCACTGTGCTAGT<br>CACACATGTCCTCACCCTAATAC	61 °C; 3.5 mM
C-X-C motif chemokine ligand 12	*Cxcl12*	NM_021704.3	TGTCAGCCACGGTGTATTT<br>CCACATACGGTAGGAGGTTTAC	61°C; 2,5 mM
atypical chemokine receptor 3	*Cxcr7*	NM_001271607.1	GACCATGTAGGCCTCAGATTAG<br>CAGCCGAGACTGGCATAAA	63 °C; 3.5 mM
interleukin 1 alpha	*Il-1α*	NM_010554.4	CCTTACACCTACCAGAGTGATTT<br>CCTTACACCTACCAGAGTGATTT	65 °C; 3 mM
interleukin 1 beta	*Il-1β*	NM_008361.4	ATGGGCAACCACTTACCTATTT<br>GTTCTAGAGAGTGCTGCCTAATG	64 °C; 3 mM
interleukin 2	*Il-2*	NM_008366.3	GCGGCATGTTCTGGATTTG<br>TGTGTTGTCAGAGCCCTTTAG	61°C; 3 mM
interleukin 6	*Il-6*	NM_031168.2	GATAAGCTGGAGTCACAGAAGG<br>TTGCCGAGTAGATCTCAAAGTG	59 °C; 3.5 mM
interleukin 14	*Il-14*	NM_001005506.3	CCTCACTTCAGCTACCTCTTAAA<br>CTACAAGTGGATGGAGGGAAAG	61 °C; 3.5 mM
-monocyte chemoattractant protein-1	*Mcp1*	NM_011333.3	CCTGGATCGGAACCAAATGA<br>CGGGTCAACTTCACATTCAAAG	62 °C; 3 mM
tumor growth factor beta	*Tgfα*	NM_031199.4	CTTTAGGAAGGACCTGGGTTG<br>GTGTGTCCAGGCTCCAAATA	66 °C; 3 mM
tumor necrosis factor alpha	*Tnfα*	NM_013693.3	GTCTCAGAATGAGGCTGGATAAG<br>CATTGCACCTCAGGGAAGAA	63 °C; 2.5 mM
Endoplasmic reticulum and oxidative stress markers				

activating transcription factor 4	*Atf4*	NM_009716.3	CCACTCCAGAGCATTCCTTTAG<br>CTCCTTTACACATGGAGGGATTAG	59 °C; 3.5 mM
binding immunoglobulin protein	*Bip*	NM_001163434.1	GAGACTGCTGAGGCGTATTT<br>CAGCATCTTTGGTTGCTTGTC	58 °C; 3.5 mM
C/EBP-homologous protein	*Chop*	NM_007837.4	TTGAGCCTAACACGTCGATTAT<br>CACTTCCTTCTGGAACACTCTC	58 °C; 3 mM
ER degradation-enhancing alpha-mannosidase-like protein	*Edem*	NM_138677.2	TGAAAGCATGTGAGGGTAGTG<br>GAGAGAAGGGAAGACAGGA-TAGA	61 °C; 3.5 mM
glucose-regulated protein 94	*Grp94*	NM_011631.1	AAGAATGAAGGAAAAACAG-GACAAAA<br>CAAATGGAGAAGATTCCGCC	58 °C; 3 mM
spliced X-box binding protein 1	*sXbp1*	NM_008934.4	GAGTCCGCAGCAGGTG<br>GTGTCAGAGTCCATGGGA	56 °C; 3 mM
glutathione peroxidase 1	*Gpx1*	NM_008160.6	GGTTCGAGCCCAATTTTACA<br>CATTCCGCAGGAAGGTAAAG	58 °C ;2.5 mM
NADPH oxidase 2	*Nox2*	NM_007807.5	ACTCCTTGGGTCAGCACTGG<br>GTTCCTGTCCAGTTGTCTTCG	62 °C; 3 mM
superoxide dismutase 1	*Sod1*	NM_011434.2	GCCTTCTGCTCGAAGTGGAT<br>GGAAGCATGGCGATGAAAGC	59 °C; 3.5 mM
superoxide dismutase 3	*Sod3*	NM_011435.3	TGGCTGATGGTTGTACCCTG<br>TGAGAAGATAGGCGACACGC	60 °C; 2.5 mM
Reference genes				
actin beta	*Actβ*	NM_007393.5	GCAAGCAGGAGTACGATGAG<br>CCATGCCAATGTTGTCTCTT	61 °C; 3.5 mM
beta 2 microglobulin	*β2m*	NM_009735.3	CCTGCAGAGTTAA-GCATGACAGT<br>TCATGATGCTTGATCACATGTCT	60 °C; 3 mM

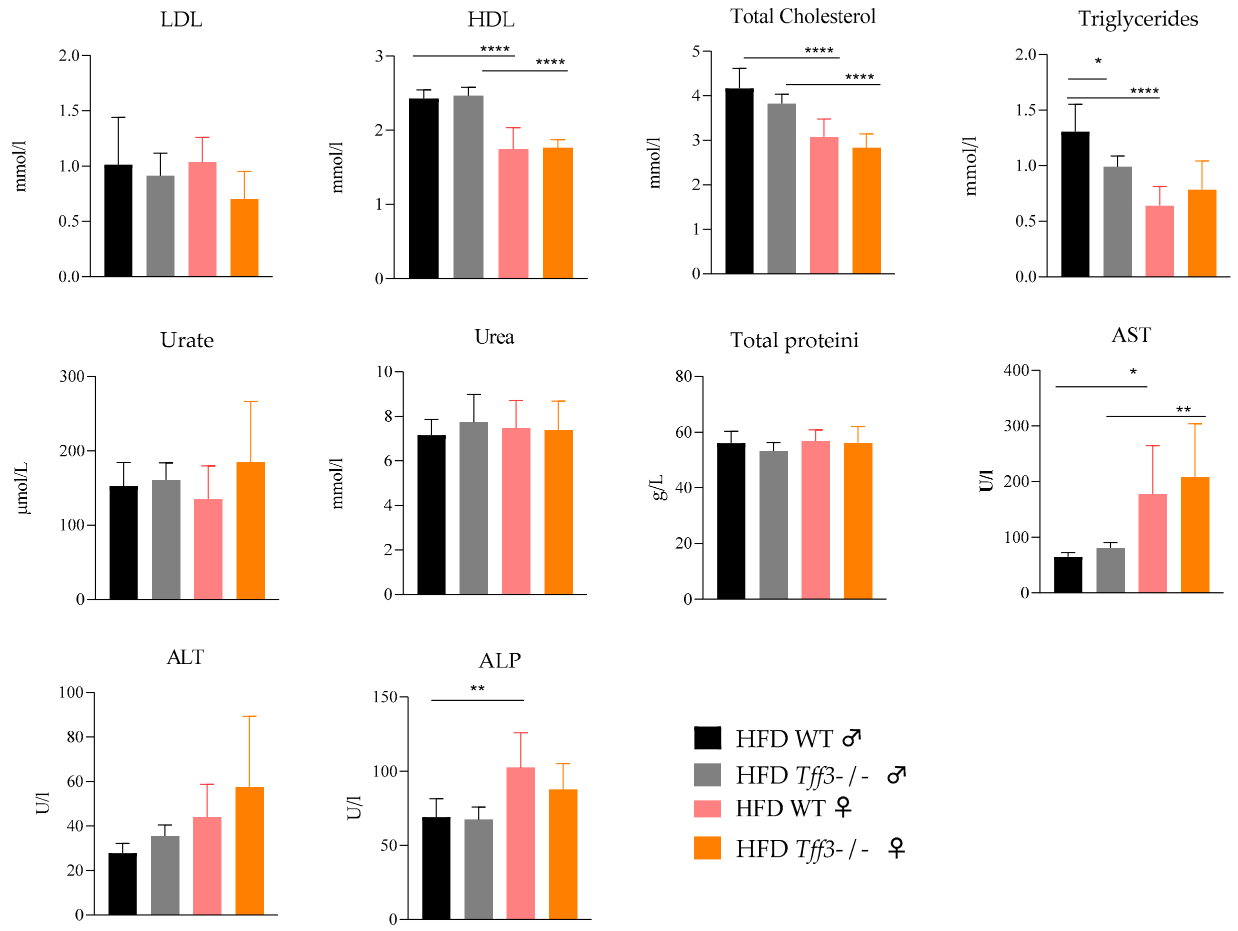

Figure S1: Effect of Tff3 deficiency on serum biochemical parameters after 9 weeks of high-fat diet. The levels of LDL (low density lipoprotein) cholesterol, HDL cholesterol (high density lipoprotein), total cholesterol, triglycerides, urate, urea, total protein, aspartate aminotransferase (AST), alanine aminotransferase (ALT) and alkaline phosphatase (ALP) were measured. Results are presented as mean value and standard deviation (n~10 per group). Two-way ANOVA followed by Bonferoni post hoc was used for statistical analysis, and significance is shown with * $p \leq 0.05$, ** $p \leq 0.01$, **** $p \leq 0.0001$

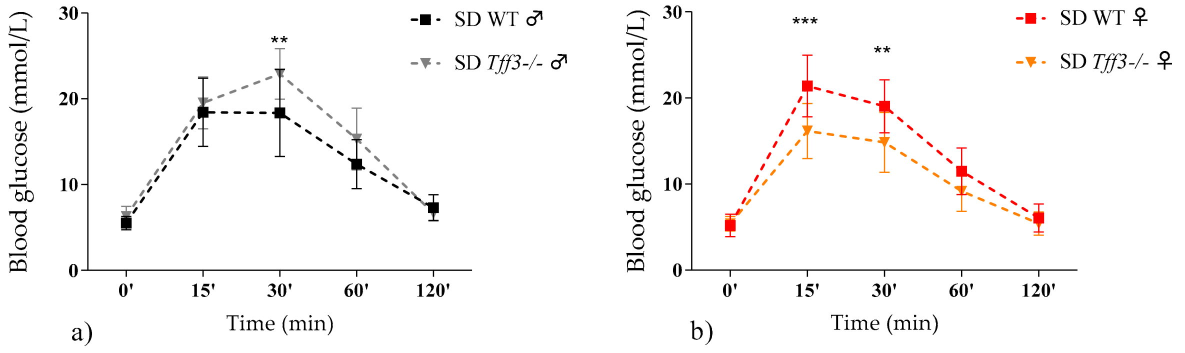

Figure S2. Intraperitoneal glucose tolerance test (IPGTT) performed on 9-week-old mice exposed to a standard diet. Blood glucose level (mmol/L) was measured at different time points; before glucose injection (0 min) and after (15, 30, 60 and 120 min). Results are presented as mean and standard deviation (SD) a) *Tff3*-/- males compared to WT males b) *Tff3*-/- females compared to WT females (n~10 mice per group). Two-way ANOVA followed by Tukey post hoc was used for statistical analysis, and significance is shown with ** p ≤ 0.01, *** p ≤ 0.001.

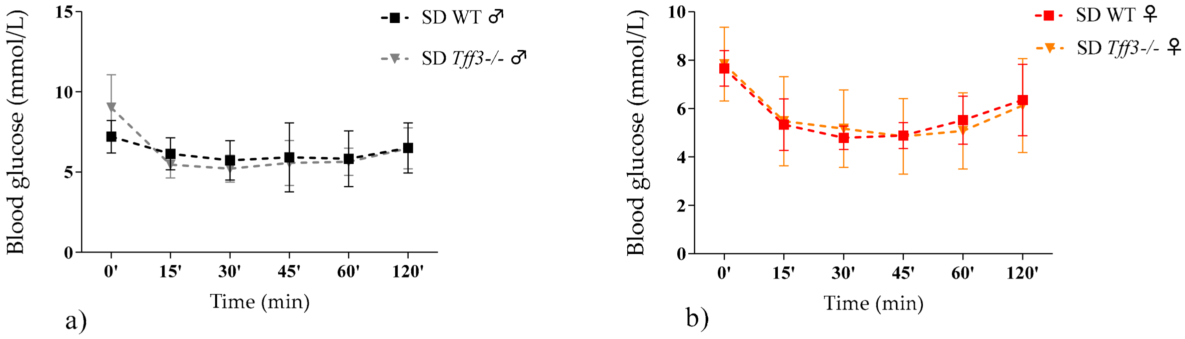

Figure S3. Intraperitoneal glucose tolerance test (IPGTT) performed on 17-week-old mice exposed to a high-fat diet for 6 weeks. Blood glucose level (mmol/L) was measured at different time intervals, before glucose injection (0 min) and after (15, 30, 60 and 120 min). Results are presented as mean and standard deviation (SD) a) *Tff3*-/- males compared to WT males b) *Tff3*-/- females compared to WT females (n~10 mice per group). Two-way ANOVA followed by Tukey post hoc was used for statistical analysis.

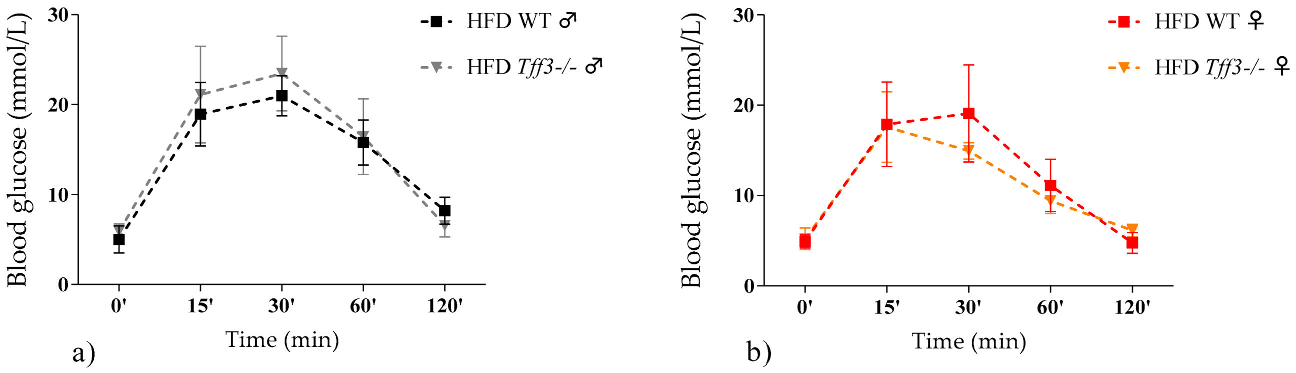

Figure S4. Intraperitoneal insulin tolerance test (IPITT) performed on 9-week-old mice exposed to standard diet. The blood glucose level (mmol/L) was measured at different time intervals, before insulin injection (0 min) and after (15, 30, 45, 60 and 120 min). Results are presented as mean and standard deviation (SD) a) *Tff3*-/- males compared to WT males b) *Tff3*-/- females compared to WT females (n~10 mice per group). Two-way ANOVA followed by Tukey post hoc was used for statistical analysis.

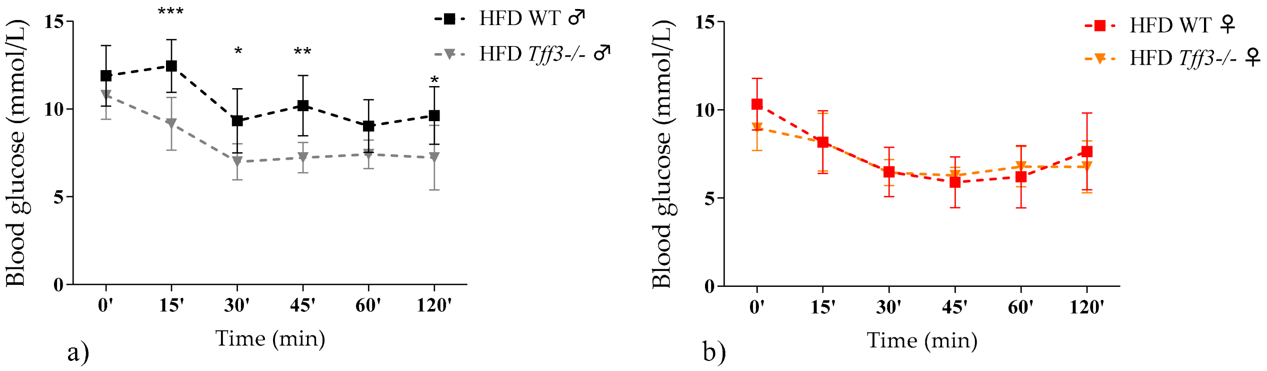

Figure S5: **Intraperitoneal insulin tolerance test (IPITT) performed on 18-week-old mice exposed to a high-fat diet for 7 weeks**. The blood glucose level (mmol/L) was measured at different time intervals, before insulin injection (0 min) and after (15, 30, 45, 60 and 120 min). Results as mean and standard deviation (SD) are shown a) Tff3-/- males compared to WT males b) Tff3-/- females compared to WT females (n~10 mice per group). Two w way ANOVA followed by Tukey post hoc was used for statistical analysis, and significance is shown with * $p \leq 0.05$, ** $p \leq 0.01$, *** $p \leq 0.001$

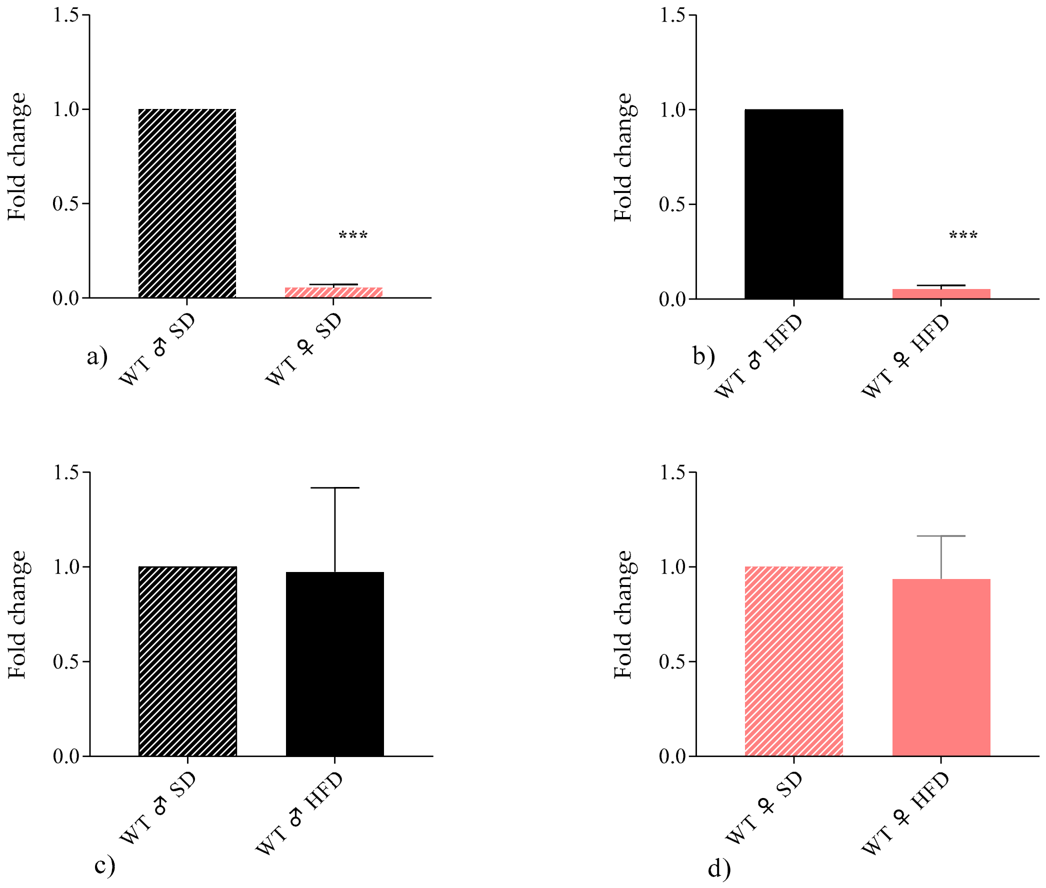

Figure S6. *Tff3* gene expression in the liver of C57BL6/N mice fed with standard and high fat diet. RNA was isolated form mouse liver tissue and geneexprerssion was monitored by qPCR method based on Sybr green detection (n=5 mice per group). Obtained Ct values were analyzed using REST© software, and results are presented as fold change and the standard error of mean value, SEM). a) expression of Tff3 in WT females on standard diet (SD) relative to WT males on SD (expresion set to 1) b) WT females on a high-fat diet (HFD) compared to WT males on a high-fat diet (HFD) (WT male HFD=1) c) WT males on a high-fat diet (HFD) in compared to WT males on a standard diet (SD) (WT males SD=1) d) WT females on a high-fat diet (HFD) compared to WT females on standard diet (SD) (WT females SD=1). Statistical significance is shown ***p ≤0.001.

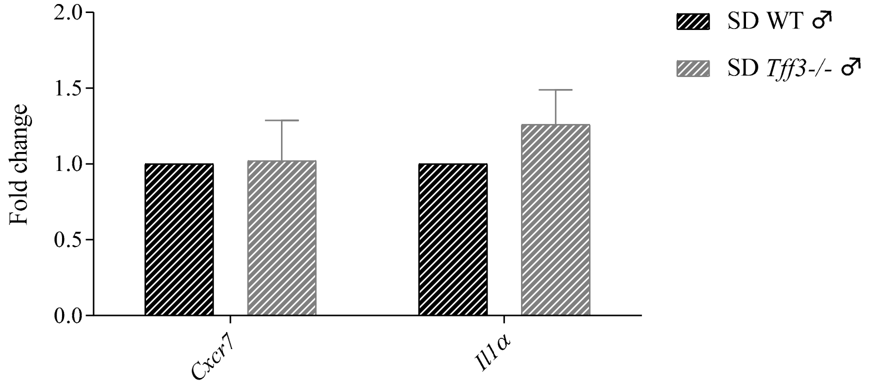

a)

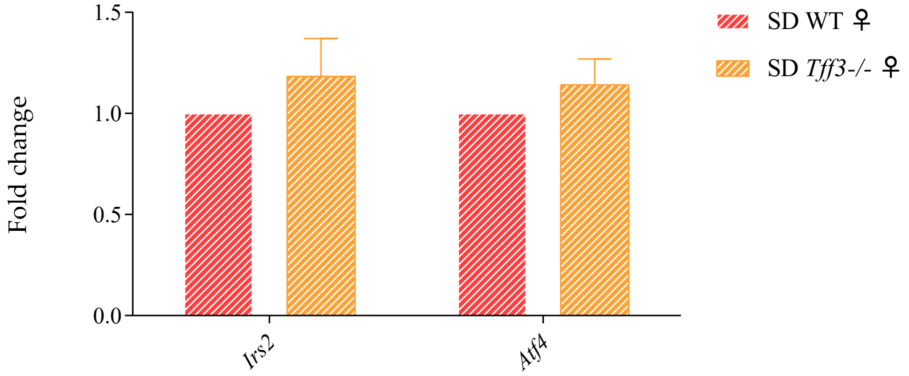

b)

Figure S7. Gene expression of markers Cxcr7, Il1α, Irs2 and Atf4 in the liver of WT and *Tff3*-/- mice exposed to SD. Mice were sacrificed liver tissue was collected, RNA was isolated and qPCR method based on Sybr green detection was used for gene expression (n=5 mice per group). The obtained Ct values were analyzed using the software REST©, and the results are presented in the form of fold change mean and standard error of the mean (SEM). a) *Tff3*-/- males compared to WT males (WT male=1) b) *Tff3*-/- females compared WT females (WT female=1).

A)

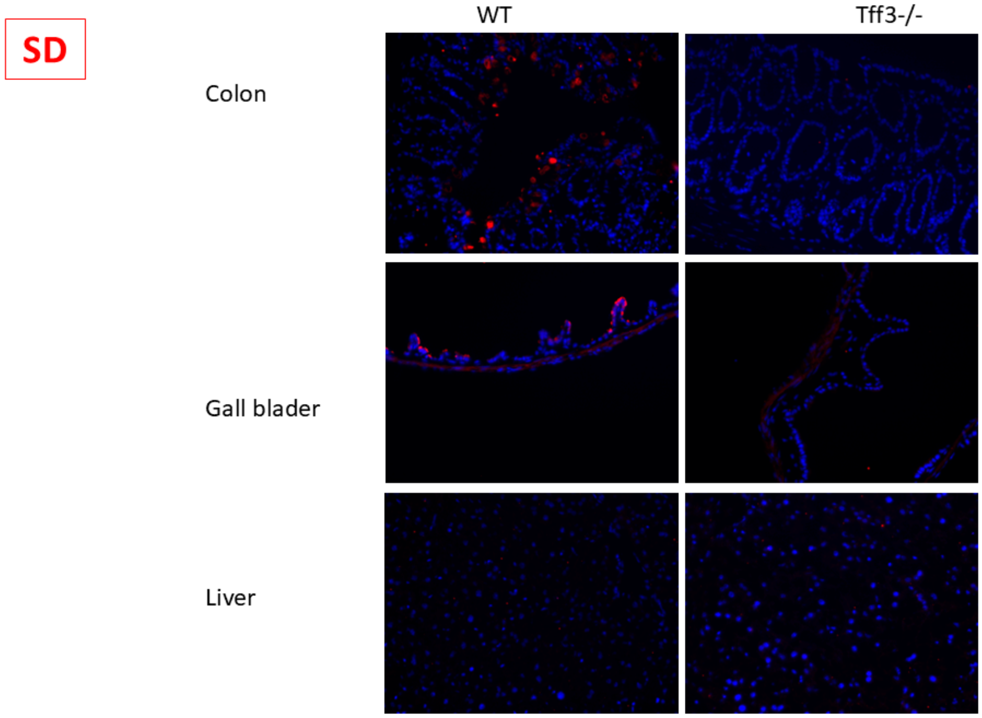

Magnification 200x

B)

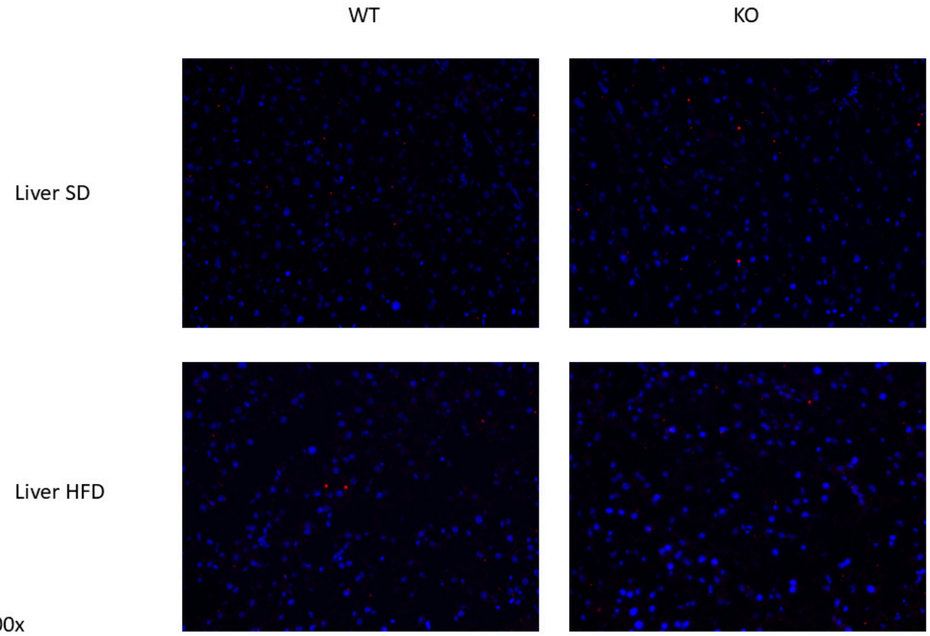

Mag: 200x

C) Expression of Tff3 in gall balder epithelia

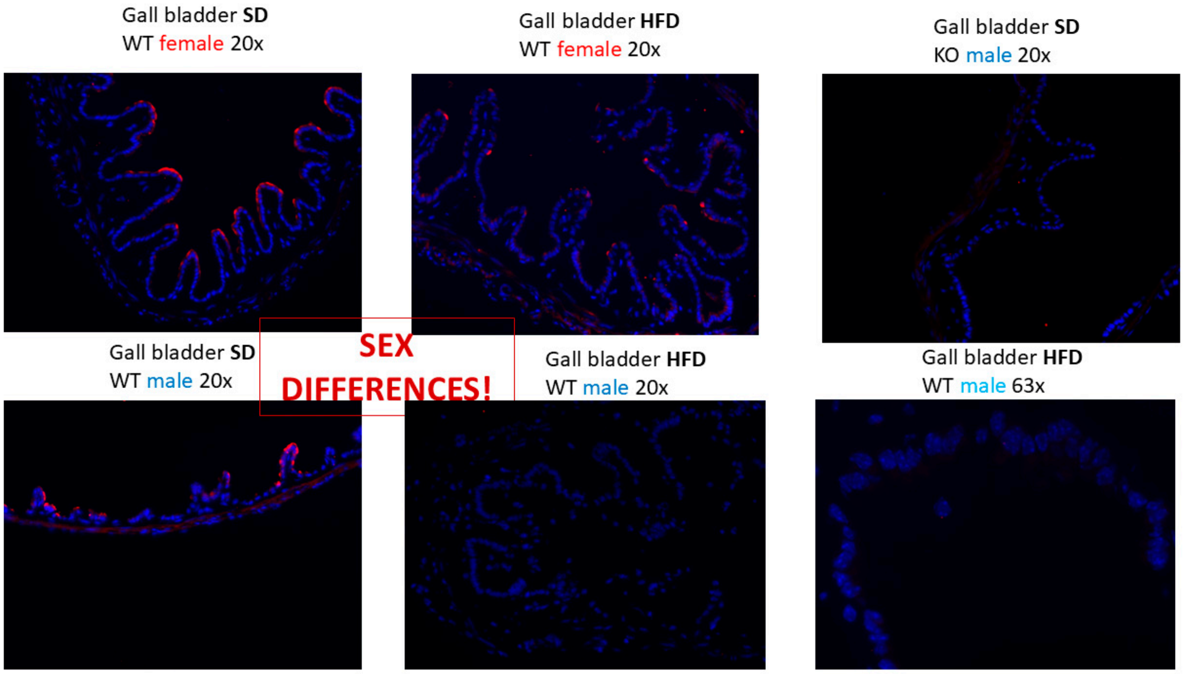

Figure S8. A) Localisation of Tff3 protein in A) colon, liver/ gall bladder of Wt and Tff3 deficient mice. B) No prominent presence of Tff3 in the liver of mice on SD and HFD; C) expression of Tff3 in gall balder is reduced upon HFD exposure.
